# Communication training for effective Goals of Patient Care conversations in acute care: An integrative review of the literature

**DOI:** 10.1017/S1478951525000264

**Published:** 2025-03-28

**Authors:** Janie Brown, Phoebe Hu-Collins

**Affiliations:** 1 Nursing, Curtin University, Perth, WA, Australia; 2 Nursing, St John of God Midland Public and Private Hospitals, Midland, WA, Australia; 3 Western Australian Group for Evidence Informed Health Practice, Curtin University, Perth, WA, Australia

**Keywords:** Communication, Goals of Patient Care, literature review, training, intervention

## Abstract

**Objectives:**

To evaluate and synthesize research that has investigated interventions to train registered health professionals to effectively communicate with patients in acute settings who are establishing their goals of care, to develop an understanding of current practices and their effectiveness.

**Design:**

Integrative review.

**Methods:**

Medline, Embase, PsycINFO, SCOPUS, CINAHL, and ProQuest, searched from the date each database was available to December 2023. Forty-seven (n = 47) research studies investigating interventions to train registered health professionals to effectively communicate with patients in acute settings who are establishing their goals of care were critically appraised for methodological quality using the Joanna Briggs Institute Quality Appraisal Framework. Minimum essential criteria and scores were agreed prior to appraisal.

**Results:**

Twenty-eight studies were excluded due to methodological quality. The 19 studies included comprised quasi experimental (n = 9), qualitative (n = 4), RCT (n = 2), text and opinion (n = 1), and mixed methods (n = 3). From these included studies 4 themes with embedded sub-themes were derived: (a) delivery of training programs, (b) clinician outcomes, (c) patient outcomes, and (d) system outcomes.

**Significance of the results:**

Communication training is essential and beneficial however its effectiveness depends on overcoming existing barriers, providing continuous learning opportunities, and embedding these into clinical practice. Addressing these factors will ensure that clinicians and healthcare organizations can improve patient and system outcomes. When clinicians and organizations prioritize regular, context-specific communication training, which promotes the use of conversation guides and available technologies, Goals of Patient Care conversations are more likely to be embedded in practice, promoting effective and patient-centered communication.

## Introduction

An understanding of patients’ goals, values, and preferences is integral in the delivery of high-quality medical care, particularly for patients with serious illnesses facing challenging treatment choices Back et al. (2019). For clinicians to gain an understanding of patients’ goals, high-quality conversations between patients, their families, and clinicians must occur (Lin et al. 2018). These conversations rely on highly effective communication skills by clinicians to communicate complex information about prognoses and treatments, elicit patient values and goals, provide support, and ensure care plans and outcomes align with patient preferences (Bernacki et al. 2019). These discussions, known as Goals of Patient Care (GoPC) conversations, have been shown to improve patient experience and quality of care, reduce costs, and improve the job satisfaction of clinicians (Back et al. 2019; Hayashi et al. 2022; Stephens et al. 2021).

GoPC conversations establish “the overarching aims of medical care for a patient that are informed by patients’ underlying values and priorities, established within the existing clinical context, and used to guide decisions about the use of or limitation(s) on specific medical interventions” (Secunda et al. 2020). This is integral to patient-centered care, where full information is provided and patients are involved in decision making, which is the standard for quality care (Jeffrey 2018). In contrast, multidisciplinary case conferences discuss patient or patients details and coordinate care among healthcare providers from across a variety of professions (Government of Western Australia, n.d.). Patients who have discussed their wishes for end-of-life care with a physician are more likely to receive care that aligns with their preferences (Clark et al. 2018; Mack et al. 2010; Wright et al. 2008), and when these conversations are discussed in a clear, empathetic way that is patient-centered, patients report greater satisfaction and rate experiences as positive (Bischoff et al. 2018; O’Connor et al. 2020). GoPC conversations are alsoassociated with less aggressive medical care near death, earlier hospice referrals, higher quality of life ratings for patients and improved bereavement outcomes for families (Bernacki and Block 2014; Tang et al. 2014; Wright et al. 2016).

Only 14.5–40% of patients with serious illness have a conversation about their goals with their clinician (Gieniusz et al. 2018; Mack et al. 2010), with even fewer conversations documented in the patient medical record (Bischoff et al. 2018). When GoPC conversations do occur, they often take place relatively late in the course of illness and neglect psychosocial, emotional, and cultural needs, with clinicians often missing opportunities to listen and respond to family members, acknowledge and address emotions, and explain treatment options (Curtis et al. 2005; Mack et al. 2012). People living with serious illnesses and their caregivers report experiencing high levels of suffering and are at risk of receiving care that is not aligned with their preferences (Committee on Approaching Death: Addressing Key End of Life Issues 2015). When GoPC conversations do not occur, physicians often misunderstand patients’ end-of-life treatment preferences (Winkler et al. 2009), and patients overestimate the benefits of life-sustaining treatments (Weeks et al. 1998). Although studies have found that patients and/or their decision makers typically prefer less aggressive care at the end of life, a concerning number of patients still receive care that does not align with their goals to minimize suffering (Gieniusz et al. 2018; Mack et al. 2010).

Clinicians often feel uncomfortable or ill-equipped to discuss end-of-life care with patients (Fulmer et al. 2018), and report high levels of moral stress when limiting life-prolonging treatment (Mehlis et al. 2018). Clinicians’ reluctance to engage in end-of-life communication has been attributed to lack of training, insufficient time, competing needs, and discomfort in communicating difficult information, responding to emotional reactions, and discussing palliative care (Committee on Approaching Death: Addressing Key End of Life Issues 2015; Granek et al. 2013; Lin et al. 2018; Waldron et al. 2016). Studies have reported relatively limited communication skills in clinicians when discussing GoPC conversations, with inappropriate use of medical jargon during, which can compromise patient understanding (O’Connor et al. 2020). Even physicians who report high confidence in being able to address patients’ concerns rarely respond empathetically when patients’ express emotions (Committee on Approaching Death: Addressing Key End of Life Issues 2015; Pollak et al. 2007). When GoPC conversations are discussed in a rushed manner or the physician is dismissive, patients report feeling fearful and invisible, and rate the experience as negative (O’Connor et al. 2020). The lack of clinician confidence and skills about palliative care and communication skills necessary to lead these conversations with patients and families is likely influenced by the absence of training in both undergraduate and graduate education (Committee on Approaching Death: Addressing Key End of Life Issues 2015; Horowitz et al. 2014; Kamal et al. 2015). These skills can be acquired through explicit training at any point in clinicians’ educational careers, and when implemented, formal teaching of palliative care and communication skills in medical school curricula and post-graduate education, including didactic and clinical learning experiences, has been shown to improve competency of clinicians (Bickel-Swenson 2007; Clayton et al. 2012; Rodenbach et al. 2020; Walczak et al. 2016).

End of life care often involves multiple, sudden and complex problems that require many healthcare professionals to be involved (Committee on Approaching Death: Addressing Key End of Life Issues 2015). This can present challenges for effective communication amongst healthcare providers, patients and their family, to effectively coordinate care to align with the patient’s preferences (Jeffrey 2018). Collaborative interdisciplinary healthcare teams can increase adoption of GoPC conversations, with Ma et al. (2021) finding that an interdisciplinary team approach improved provider perception of goals of care uptake. When provided training that encouraged nurses and social workers to initiate conversations to educate patients on goals of care discussions, physicians and advanced practice providers would follow up their conversations, confirm patients’ wishes, and sign-related orders (Ma et al. 2021). Similarly, Stephens et al. (2021) found that interdisciplinary educational environments improved training experiences and identified diversity of participants’ perspectives to be a key learning point, reporting improvements in communication skills with patients and also interprofessional team members.

Goal-concordant care also relies on systems to ensure information can be documented and accessed when needed. Benton (2017) found that GoPC conversations were more likely to occur when a formal system was in place for assessing patients’ end-of-life wishes and goals of care, suggesting that systematic interventions are also necessary in facilitating effective communication between clinicians and their patients. This requires a significant shift in the organization of healthcare systems, leadership recognition and involvement, training for providers, and support to manage systems and roles across disciplines (Ma et al. 2021). When patients’ preferences are poorly or not documented, they are at greater risk of receiving care that does not align to their wishes (Heyland et al. 2013). Heyland et al. (2013) found that nearly 70% of documented orders were discordant with patients’ expressed preferences for end-of-life, despite documentation occurring shortly after conversations. System-level barriers, such as lack of accessible documentation, lack of training to navigate systems, and storing and updating patient information across time and locations may contribute to difficulties providing goal-concordant care (Heyland et al. 2013; Lakin et al. 2016; Turley et al. 2016). Lakin et al. (2016) found that physicians reported low confidence in finding or using documentation in the Electronic Medical Records to care for patients, suggesting clear guidelines for GoPC conversations need to be embedded within hospital policies and into communication skills training (O’Connor et al. 2020).

To improve communication skills training about end-of-life care, several postgraduate education programs have been developed. These typically have been designed for physicians or residents, with relatively few non-physician professionals participating (Bays et al. 2014). Training programs have increasingly utilized experiential learning and skills practice through simulation, where clinicians role play communication with simulated patients (Cannone et al. 2019). Practicing communication skills in a high fidelity but low risk simulated environment aims to prepare clinicians for real life encounters while decreasing learner stress and fostering a safe learning environment (Back et al. 2019). As part of skills practice, training programs typically also include a feedback element to encourage clinicians’ development of self-awareness and perceptions of their own or others’ emotions, attitudes, and underlying beliefs that may impact communication (Back et al. 2019; Thomas et al. 2014). Delivery of communication skills training is primarily through in-person workshops, though recently virtual training programs have been developed to increase reach and observe social distancing requirements (Crossman et al. 2021; Uemura et al. 2024). Back et al. (2019) outline a number of established communication training workshops that vary greatly in curriculum, format, and target populations. These focus on using a scripted conversation guide (Ariadne Labs 2024), advanced care planning (Choices R 2024), foundational communication skills (Center to Advance Palliative Care 2024), flexible use of communication skills (VITALtalk 2024), interprofessional skills (American Association of Colleges of Nursing 2024), and providing an overview of communication skills (Back et al. 2019; Northwestern University 2024).

Despite recommendations for mandatory communication skills training at undergraduate and postgraduate levels (references), almost none of the established programs or models have been widely disseminated in health professional accreditation requirements or training curricula (Back et al. 2019; Gilligan et al. 2017). Although there has been an increase in palliative care training programs in the last 20 years, teaching methods vary greatly between programs, with trainees and leaders often rating current education as inadequate (Albert et al. 2023). Overall, there has been a lack of widely adopted interdisciplinary pedagogical models based on clear theoretical frameworks that can be tailored to teach a broad spectrum of difficult conversations. As such, this review explores research that has investigated interventions to train health professionals to effectively communicate with patients in acute settings who are establishing their goals of care.

## Methods

### Aim

This integrative review evaluates and synthesises research that investigated interventions to train registered health professionals to effectively communicate with patients in acute settings who are establishing their goals of care. This review describes current uni- and multidisciplinary training interventions and their clinician, patient, and system outcomes.

### Design

**Figure 1.**
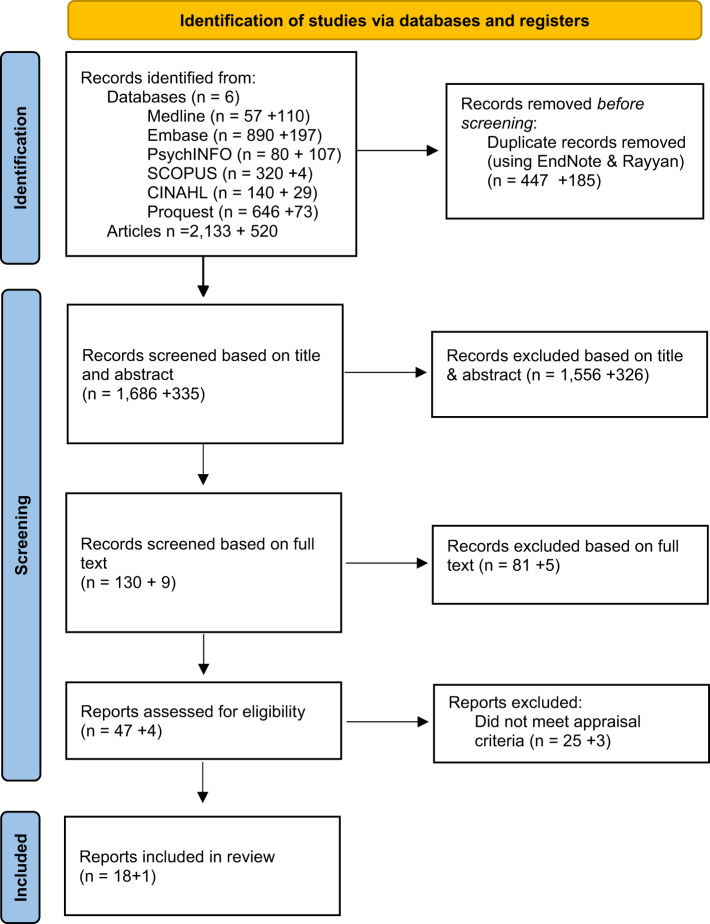
The + indicates updated search conducted in November–December 2023, in addition to original search in February 2023. *From:* **Page MJ, Mckenzie JE, Bossuyt PM, *et al.*** (2021) The PRISMA 2020 statement: An updated guideline for reporting systematic reviews. *BMJ* **372**, n71. https://doi.org/10.1136/bmj.n71. For more information, visit: http://www.prisma-statement.org/.

This review used the Whittemore and Knafl (2005) Integrative Review methodology and followed the Preferred Reporting Items for Systematic Reviews (PRISMA) checklist (Page et al. 2021) (Fig. 1). This approach allowed the analysis and synthesis of both empirical (qualitative and quantitative) and theoretical literature related to GoPC conversations. Following this approach, problem identification, literature search, data evaluation, data analysis and presentation were completed. Problem identification involved identifying the aim of the review, target population, intervention, outcome. A literature search was undertaken, and inclusion criteria was formulated. Relevant reports were critically appraised using the Joanna Briggs Institute Methodological Appraisal tools (Aromataris et al. 2024), and data relevant to the aim of the review were extracted and is presented in table format. Themes and subthemes were identified and are presented in a table format with an accompanying narrative discussion. Finally, conclusions to inform practice, policy and education and further research are presented (Whittemore and Knafl 2005).

### Search methods

A search strategy was developed with the assistance of the Faculty of Health librarian. A concept grid was used to determine the likely subject headings and keywords. Appropriate combining search terms were explored.

The search terms include variations of the following keywords and phrases; doctor* or physician* or clinician* or nurse* or hospitalist* or “health* professional*” or “allied health*” or physiotherapist* or “occupational therapist*” or fellow* or “speech pathologist*” or “social work*” or oncologist*, intervention* or program* or training* or VitalTalk* or workshop* or course*, Communication* or conversation*, goal* ADJ3 care or goc* or gopc* or “treatment* goal*” or “treatment* choice*” or “end-of-life conversation*” or “serious illness conversation*” or “difficult conversation*,” and hospital* or “acute setting*” or “emergency* department*” or “intensive care unit*” or center* or outpatient*

The following databases were searched; CINAHL, Embase, PsycINFO, Medline (Ovid), Scopus, and ProQuest.

Inclusion criteria captured qualitative, quantitative and mixed methods, and text and opinion studies, explicitly investigating communication skills training for registered health professionals conducting GoPC conversations, published in peer-review journals. Exclusion criteria were studies that included undergraduate students, non-registered health professionals (for example, ENs or AINs), or outpatient settings.

### Search outcomes

The initial literature search was conducted in February 2023, with an updated search conducted in December 2023. The initial search identified 2,133 records; 223 were duplicates identified using EndNote (The EndNote Team 2013) and Rayyan (Ouzzani et al. 2016) leaving 1,910 for possible inclusion (Fig. 1). Titles and abstracts of the remaining records were reviewed (author initials to be included after peer review) and 1,786 were excluded. This left 124 records for full-text review (author initials to be included after peer review), after which a further 81 records were excluded leaving 43 reports for quality appraisal (author initials to be included after peer review). The second search identified 520 new records published between February 2023 and December 2023; 185 were duplicates EndNote (The EndNote Team 2013) and Rayyan (Ouzzani et al. 2016) leaving 335 for possible inclusion. Titles and abstracts of the remaining records were reviewed (author initials to be included after peer review) and 326 were excluded. This left 9 records for full-text review (author initials to be included after peer review), after which a further 5 records were excluded leaving 4 reports for quality appraisal (author initials to be included after peer review). Records were input into Retraction Watch (The Center for Scientific Integrity 2018) on 14/05/2024 to ensure no records had been retracted.

### Quality appraisal

The data evaluation stage was completed using the Joanna Briggs Institute (JBI) Quality Appraisal Framework (2017). Studies which met the inclusion criteria based on title and abstract review were read independently in full by two authors to confirm eligibility and to conduct quality appraisal for methodological quality using the appropriate The JBI (2017) quality appraisal tools. Six tools were used; the checklists for analytical cross-sectional studies, for qualitative research, for quasi-experimental studies (non-randomized experimental studies), systematic reviews, text and opinion, and for randomized controlled trials (RCTs; The Joanna Briggs Institute, 2017). There is no specific tool to evaluate descriptive studies, so the checklist for analytical cross-sectional studies was adapted to review descriptive studies. Mixed methods studies were appraised in two parts using an appropriate quantitative checklist and the checklist for qualitative research. Quality appraisal criteria were discussed, and consensus was reached upon which criteria were essential and any modifications and considerations in the assessment of criteria for each tool. To be included in the review, studies had to meet all essential criteria and be in two points of the total achievable score (Tables 1–8). Where criterion was not applicable to a particular study, the total achievable score was reduced. Following quality appraisal, a further 25 from the initial search, and three publications from the second search did not meet the required level of quality, leaving a total of 19 for review.

**Table 1.** Critical appraisal of eligible systematic review and research synthesis – search one only

Citation	Q1 Is the review question clearly and explicitly stated? Essential	Q2 Were the inclusion criteria appropriate for the review question? Essential	Q3 Was the search strategy appropriate? Essential	Q4 Were the sources and resources used to search for studies adequate? Essential	Q5 Were the criteria for appraising studies appropriate? Essential	Q6 Was critical appraisal conducted by two or more reviewers independently? Essential	Q7 Were there methods to minimize errors in data extraction? Essential	Q8 Were the methods used to combine studies appropriate? Essential	Q9 Was the likelihood of publication bias assessed? Desirable but not essential	Q10 Were recommendations for policy and/or practice supported by the reported data? Essential	Q11 Were the specific directives for new research appropriate? Essential	Q12 Peer review journal? Essential	Score	Include
Bennett and O’Conner-Von (2020)	✓	✓	✓	✓	✓	U	U	U	✗	✓	✓	✓	8/12	No^1^

1 Did not meet an essential criterion.

**Table 2.** Critical appraisal of eligible randomized controlled trials – search one only

Citation	Q1 Was true randomization used for assignment to treatment groups? Desirable but not essential	Q2 Was allocation to treatment concealed? Desirable but not essential	Q3 Were treatment groups similar at baseline? Essential	Q4 Were participants blind to treatment assignment. Desirable but not essential based on whether blinding was possible	Q5 Were those delivering treatment blind to treatment assignment? Desirable but not essential based on whether blinding was possible	Q6 Were outcome assessors blind to treatment assignment? Desirable but not essential	Q7 Were treatment groups treated identically other than the intervention of interest? Desirable but not essential	Q8 Was follow up complete and if not, were differences between groups in terms of their follow up adequately described and analyzed? Desirable but not essential	Q9 Were participants analyzed in the groups to which they were randomized? Essential	Q10 Were outcomes measured in the same way for treatment groups? Essential	Q11 Were outcomes measured in a reliable way? Essential It was agreed that pilot studies, underpowered studies or studies with a significant attrition rate will be included	Q12 Was appropriate statistical analysis used? Essential	Q13 Was the trial design appropriate, and any deviations from the standard RCT design (individual randomization, parallel groups) accounted for in the conduct and analysis of the trial? Essential	Q14 Peer review journal? Essential	Q15 HREC approval? Essential	Score	Include
Annadurai et al. (2021)	U	N/A	✓	N/A	N/A	✓	U	✓	✓	✓	✓	✓	✓	✓	✓	10/12	Yes
Bernacki et al. (2019)	U	U	✓	U	✗	U	U	✓	✓	✓	✓	✓	U	✓	✓	8/15	No^1^
Bowen et al. (2020)	✓	U	✓	✗	✗	✓	✓	✓	✓	✓	✓	✓	U	✓	✓	11/15	No^1^
Epstein et al. (2017)	✓	U	✓	✓	N/A	✓	✓	✓	✓	✓	✓	✓	✓	✓	✓	13/14	Yes
Goldstein et al. (2019)	U	N/A	U	N/A	N/A	U	✓	✓	✓	✓	U	✓	✓	✓	✓	8/12	No^1^
Paladino et al. (2019)	U	U	✓	✓	✗	✓	U	✓	✓	✓	✓	✓	✓	✓	✓	11/15	No^1^
Paladino et al. (2020a)	U	U	✓	✓	✗	✓	✓	✓	✓	✓	✓	✓	✓	✓	✓	12/15	No^1^
Pollak et al. (2019)	✓	✗	✗	✗	✗	✗	U	U	✓	✓	U	✓	U	✓	✓	6/15	No^1^

1 Did not meet the threshold and an essential criterion.

**Table 3.** Critical appraisal of eligible quasi-experimental studies – searches one and two

Citation – search 1	Q1 Is it clear in the study what is the “cause” and what is the “effect” (i.e there is no confusion about which variable comes first). Essential		Q2 Were the participants included in any comparisons similar? Desirable but not essential		Q3 Were the participants included in any comparisons receiving similar treatment/care, other than the exposure or intervention of interest? Desirable but not essential		Q4 Was there a control group? Desirable but not essential – Agreed that a comparison or control was accepted.		Q5 Were there multiple measurements of outcomes both pre and post the intervention/exposure? Desirable but not essential Agreed that one pre and one post measurement was accepted		Q6 Was follow up complete and if not, were differences between groups in terms of their follow up adequately described and analyzed? Desirable but not essential		Q7 Were the outcomes of participants included in any comparisons measured in the same way? Desirable but not essential		Q8 Were outcomes measures in a reliable way? Essential		Q9 Was appropriate statistical analysis used? Essential. It was agreed that pilot studies, underpowered studies or studies with a significant attrition rate will be included		Q10 Peer review journal? Essential	Q11 HREC approval Essential	Score	Include	
Ajayi et al. (2019)	✓		✓		N/A		✗		✓		N/A		N/A		U		✓		✓	✗	5/8	No^1^	
Baer and Weinstein (2013)	✓		N/A		N/A		✗		✓		N/A		N/A		U		U		✓	✗	3/7	No^1^	
Banerjee et al. (2017)	✓		N/A		N/A		✗		✓		N/A		N/A		✓		✓		✓	✓	6/7	Yes	
Berlacher et al. (2017)	✓		N/A		N/A		✗		✓		N/A		N/A		U		✓		✓	✓	5/7	No^1^	
Berns et al. (2017)	✓		N/A		N/A		✗		✓		N/A		N/A		✓		✓		✓	✓	6/7	Yes	
Bigelow et al. (2023)	✓		N/A		N/A		✗		✓		N/A		N/A		U		✓		✓	✓	5/7	No^1^	
Billie and Letizia (2020)	✓		N/A		N/A		✗		✓		N/A		N/A		U		✓		✓	✓	5/7	No^1^	
Boss et al. (2013)	✓		N/A		N/A		✗		✓		N/A		N/A		✓		U		✓	✓	5/7	No^1^	
Casey et al. (2022)	✓		N/A		N/A		✗		✓		N/A		N/A		✓		✓		✓	✓	6/7	Yes	
Chatterjee et al. (2022)	✓		N/A		N/A		✗		✓		N/A		N/A		✓		✓		✓	✓	6/7	Yes	
Childers and Arnold (2018)	✓		U		U		✓		✓		U		✓		✓		✓		✓	✓	8/11	No^2^	
Fettig et al. (2022)	✓		N/A		N/A		✗		✓		N/A		N/A		✓		✓		✓	✓	6/7	Yes	
Frydman et al. (2021)	✓		N/A		N/A		✗		✓		N/A		N/A		U		✓		✓	✓	5/7	No^1^	
Haley et al. (2017)	✓		N/A		N/A		✗		✗		N/A		N/A		✓		✓		✓	✓	5/7	Yes	
Lakin et al. (2021)	✓		U		✓		✓		✗		U		✓		✓		✓		✓	✓	8/11	No^2^	
Lakin et al. (2017)	✓		U		U		✓		✗		✓		✓		✓		✓		✓	✓	8/11	No^2^	
Moody et al. (2020)	✓		N/A		N/A		✗		✓		N/A		N/A		✓		✓		✓	✓	6/7	Yes	
Ouchi et al. (2023b)	✓		N/A		N/A		✗		✓		N/A		N/A		✓		✓		✓	✓	6/7	Yes	
Runkle et al. (2008)	✓		N/A		N/A		✗		✓		N/A		N/A		U		✓		✓	✓	5/7	No^1^	
Smith (2017)	✓		N/A		N/A		✗		✓		N/A		N/A		✓		✓		✓	✓	6/7	Yes	
Citation – search 2																							
Kumar et al. (2023)	✓		N/A		N/A		N/A		✗		N/A		N/A		U		U		✓	✓	3/8	No^1^	
Aaronson et al. (2023)	✓	✓	✓	✓	✓	✗	✗	✓	✓	✓	✓	N/A	✓	N/A	N	U	✓	✓	✓	✓	✓	✓	U

1 Did not meet an essential criterion and the threshold.

2 Did not meet the threshold.

**Table 4.** Critical appraisal of eligible analytical cross-sectional study – search two only

Citation – search 2	Q1 Were the criteria for inclusion in the sample clearly defined? Essential	Q2 Were the study subjects and the setting described in detail? Essential	Q3 Was the exposure measured in a valid and reliable way? Essential	Q4 Were objective, standard criteria used for measurement of the condition? Essential	Q5 Were confounding factors identified? Essential	Q6 Were strategies to deal with confounding factors stated? Essential	Q7 Were the outcomes measured in a valid and reliable way? Essential	Q8 Was appropriate statistical analysis used? Essential	Q10 Peer review journal? Essential	Q11 HREC approval Essential	Score	Include
Rivet et al. (2023)	✓	✓	✓	N/A	✓	✗	✓	✓	✓	N/A	8/9	No^1^

1 Did not meet an essential criterion.

**Table 5.** Critical appraisal of eligible qualitative research – searches one and two

Citation – search 1	Q1 Is there congruity between the stated philosophical perspective and the research? Essential – it was agreed that a demonstrated sound qualitative approach rather than a stated philosophical perspective would be satisfactory	Q2 Is there congruity between the research methodology and the research questions or objectives? Essential	Q3 Is there congruity between the research methodology and the methods used to collect the data? Essential	Q4 Is there congruity between the research methodology and the representation and analysis of the data? Essential	Q5 Is there congruity between the research methodology and the interpretation of the results? Essential	Q6 Is there a statement locating the researcher culturally or theoretically? Desirable but not essential	Q7 Is the influence of the researcher on the research, and vice-versa addressed? (Includes evidence of measure to check) trustworthiness) Essential if qualitative only, desired if mixed	Q8 Are participants and their voices adequately represented? Essential	Q9 Is the research ethical according to current criteria or, for recent studies, and is there evidence of ethical approval by an appropriate body? Essential	Q10 Do the conclusions drawn in the research report flow from the analysis, or interpretation, of the data? Essential	Q11 Peer review journal? Essential	Score	Comment
Day et al. (2022)	✓	✓	✓	✓	✓	✓	✓	✓	✓	✓	✓	11/11	Yes
Garcia et al. (2023)	✓	✓	✓	✓	✓	✗	✗	✓	✓	✓	✓	9/11	No^1^
Geerse et al. (2019)	✓	✓	✓	✓	✓	✗	✓	✓	✓	✓	✓	10/11	Yes
Lagrotteria et al. (2021)	✓	✓	✓	✓	✓	✗	✓	✓	✓	✓	✓	10/11	Yes
Lakin et al. (2019)	✓	✓	✓	✓	✓	✗	✗	✓	✓	✓	✓	9/11	No^1^
**Citation – search 2**													
Doherty et al. (2023)	✓	✓	✓	✓	✓	✗	✓	✓	✓	✓	✓	10/11	Yes

1 Did not meet an essential criterion.

**Table 6.** Critical appraisal of eligible text and opinion studies – searches one and two

Citation – search 1	Q1 Is the source of the opinion clearly identified? Essential	Q2 Does the source of opinion have standing in the field of expertise? Essential	Q3 Are the interests of the relevant population the central focus of the opinion? Essential	Q4 Is the stated position the result of an analytical process, and is there logic in the opinion expressed? Essential	Q5 Is there reference to the extant literature? Essential	Q6 Is any incongruence with the literature / sources logically defended? Essential	Q7 Peer review journal? Essential	Score	Include
Wolfe et al. (2016)	✓	✓	✓	✓	✓	N/A	Yes	6/6	Yes
**Citation – search 2**									
Seevaratnam et al. (2024)	✓	✓	✓	U	✓	N/A	Yes	5/6	No^1^

1 Did not meet an essential criterion.

**Table 7.** Critical appraisal of eligible mixed methods studies (qualitative and RCT) – search one only

Citation	Q1 Is there congruity between the stated philosophical perspective and the research? Essential – it was agreed that a demonstrated sound qualitative approach rather than a stated philosophical perspective would be satisfactory	Q2 Is there congruity between the research methodology and the research questions or objectives? Essential	Q3 Is there congruity between the research methodology and the methods used to collect the data? Essential	Q4 Is there congruity between the research methodology and the representation and analysis of the data? Essential	Q5 Is there congruity between the research methodology and the interpretation of the results? Essential	Q6 Is there a statement locating the researcher culturally or theoretically? Desirable but not essential	Q7 Is the influence of the researcher on the research, and vice-versa addressed? (Includes evidence of measure to check trustworthiness) Essential if qualitative only, desired if mixed	Q8 Are participants and their voices adequately represented? Essential	Q9 Is the research ethical according to current criteria or, for recent studies, and is there evidence of ethical approval by an appropriate body? Essential	Q10 Do the conclusions drawn in the research report flow from the analysis, or interpretation, of the data? Essential	Q1	Q2	Q3	Q4	Q5	Q6	Q7	Q8	Q9	Q10	Q11	Q12	**Q13**
Aaronson et al. (2023)	✓	✓	✓	✓	✓	✗	✗	✓	✓	✓	✓	N/A	✓	N/A	N	U	✓	✓	✓	✓	✓	✓	U

**Table 8.** Critical appraisal of eligible mixed methods studies (qualitative and quasi-experimental) – search one only

Citation – search 1	Q1 Is there congruity between the stated philosophical perspective and the research? Essential – it was agreed that a demonstrated sound qualitative approach rather than a stated philosophical perspective would be satisfactory	Q2 Is there congruity between the research methodology and the research questions or objectives? Essential	Q3 Is there congruity between the research methodology and the methods used to collect the data? Essential	Q4 Is there congruity between the research methodology and the representation and analysis of the data? Essential	Q5 Is there congruity between the research methodology and the interpretation of the results? Essential	Q6 Is there a statement locating the researcher culturally or theoretically? Desirable but not essential	Q7 Is the influence of the researcher on the research, and vice-versa addressed? (Includes evidence of measure to check trustworthiness) Essential if qualitative only, desired if mixed	Q8 Are participants and their voices adequately represented? Essential	Q9 Is the research ethical according to current criteria or, for recent studies, and is there evidence of ethical approval by an appropriate body? Essential	Q10 Do the conclusions drawn in the research report flow from the analysis, or interpretation, of the data? Essential	Q1	Q2	Q3	Q4	Q5	Q6	Q7	Q8	**Q9**
Benesch et al. (2022)	✓	✓	✓	✓	✓	✗	✗	✓	✓	✓	✓	N/A	N/A	✗	✓	N/A	N/A	U	✓
Clarke et al. (2023)	✓	✓	✓	✓	✓	✗	✗	✗	✗	✗	✓	N/A	N/A	✗	✓	N/A	N/A	U	✓
Crossman et al. (2021)	U	✓	✓	✓	✓	✗	✗	✓	✓	✓	✓	N/A	N/A	✗	✗	N/A	N/A	U	✓
Drenner, (2022)	N/A	N/A	N/A	N/A	N/A	N/A	N/A	N/A	N/A	N/A	✓	N/A	N/A	✗	✗	N/A	N/A	U	✓
Gagliardi and Morassaei (2019)	✓	✓	✓	✓	✓	✗	✗	✓	✗	✓	✓	N/A	N/A	✗	✓	N/A	N/A	U	✓
Kruser et al. (2017)	✓	✓	U	✓	✓	✗	✗	✓	✓	✓	✓	N/A	N/A	✗	✗	N/A	N/A	✓	✓
Milic et al. (2015)	✓	✓	✓	✓	✓	✗	✗	✓	✓	✓	✓	N/A	N/A	✗	✓	N/A	N/A	U	✓
Paladino et al. (2020b)	✓	✓	✓	✓	✓	✗	✗	✓	✓	✓	✓	N/A	N/A	✗	✗	N/A	N/A	U	✓
Pasricha et al. (2020)	✓	✓	✓	✓	✓	✗	✗	✓	✓	✓	✓	N/A	N/A	✗	✗	N/A	N/A	✓	✓
Wasp et al. (2021)	✓	✓	✓	✓	✓	✗	✗	✓	✓	✓	✓	N/A	N/A	✗	✓	N/A	N/A	✗	✓
White, White et al. (2022)	✓	✓	✓	✓	✓	✗	✗	✓	✓	✓	✓	N/A	N/A	✗	✓	N/A	N/A	U	✓

### Data extraction and synthesis

Data about the text and studies’ sample sizes, interventions, and measures were extracted (see Supplementary file 1). Results data from each study and one text were also extracted (see Supplementary file 1). As data were extracted (see Supplementary file 1), consistent patterns and themes across the sources, for example, similar statistical outcomes, words, phrases or concepts that were reported in the studies’ results or key findings were tabulated. Subthemes were identified and then collapsed into themes (data analysis stage) according to the review aim.

## Results

### Included studies

The 19 included studies (Table 9) were published over a 7-year period, from 2016 to 2023. Four were qualitative (Day et al. 2022; Doherty et al. 2023; Geerse et al. 2019; Lagrotteria et al. 2021), nine were quasi-experimental (Banerjee et al. 2017; Berns et al. 2017; Casey et al. 2022; Chatterjee et al. 2022; Fettig et al. 2022; Haley et al. 2017; Moody et al. 2020; Ouchi et al. 2023; Smith 2017), two were randomized controlled trials (Annadurai et al. 2021; Epstein et al. 2017), three were mixed methods (Aaronson et al. 2023; Kruser et al. 2017; Pasricha et al. 2020), and one was a text article (Wolfe et al. 2016). Studies originated in the United States of America (Aaronson et al. 2023; Annadurai et al. 2021; Banerjee et al. 2017; Berns et al. 2017; Casey et al. 2022; Chatterjee et al. 2022; Epstein et al. 2017; Fettig et al. 2022; Geerse et al. 2019; Haley et al. 2017; Kruser et al. 2017; Moody et al. 2020; Ouchi et al. 2023; Pasricha et al. 2020; Smith 2017; Wolfe et al. 2016) and Canada (Day et al. 2022; Doherty et al. 2023; Lagrotteria et al. 2021). Sample sizes ranged from 3 to 342 participants (qualitative studies 3–25 and quantitative studies 6–342).

**Table 9.** Summary of included studies

Author, year and country	Aim/purpose	Setting or context	Design	Sample	Intervention	Measure/s	Results	Themes
Aaronson et al. (2023), USA	To assess the acceptability and feasibility of a scripted palliative care communication intervention in the ED directed by social workers.	Academic medical center	Mixed methods RCT and qualitative	Patients (n = 65) Social workers in focus groups (n = 3)	SIC script for GOPC conversations. Social workers received training on the script’s content and use, a simulation with a palliative care physician and a patient actor. Before study start, each social worker administered the conversation with one patient volunteer. Weekly or biweekly meetings with the ED social workers and research staff, including an ED physician, an expert in serious illness communications, and a palliative care social worker, were ongoing during the study period to help troubleshoot logistics and concerns that arose about SIC interactions.	Quant: Quality of Serious Illness Conversation (QSIC) to assess the degree to which patients find conversations acceptable. Quant: Feasibility, measured by percentage of completion and follow up rate. Qual: thematic analysis of social worker focus groups analyzed using a inductive approach	Quant: Social workers were able to complete a SIC for 66.1% of patients. 88% follow-up rate combined across both study groups Quant: Social workers consistently administered 95% of the intervention components. Quant: Of patients who received the intervention (n = 43) and had completed follow-up assessments (n = 38), 77% reported that they appreciated the social worker bringing up their personal goals for the future; 72% reported they appreciated the social worker asking about their fears and worries; and 81% reported they liked the way the conversation about their illness was set up. Qual: Themes emerged related to the work being gratifying, meaningful, and thought provoking, and a scope of work that aligned well with their training:	1, 2, 4
Annadurai et al. (2021), USA	To assess the impact of a communication training and coaching intervention (INT) for oncologists during GoC discussions.	Academic medical center, community hospital, municipal hospital, and community-based rural hospital.	RCT	Solid tumor oncologists n = 11 were randomized to the INT group and n = 11 to the UC group.	SPIKES. Communication training for oncologists based on VitalTalk. Clinician training included a one hour didactic, and two hours of roleplay.	Scoring of pre/post audio recording of clinical encounter to assess comskills. by three blinded coders trained as a VitalTalk communication skills teachers. Surveys of clinician comfort levels	Between trial arms, there was only one significant difference in comfort level with certain communication skills: comfort level with discussing prognosis with advanced cancer patients, with 73% (n = 7) INT oncologists and 18% (n = 8) of UC oncologists reported being comfortable doing so ( p = 0.03). There were no significant differences in prior communication skills training In the post-INT audiotaped visits, INT oncologists were significantly more likely to elicit patient values (55% vs. 0%; p = 0.01). There was no significant difference in the use of any other skills between pre- and post-INT assessments among UC and INT oncologists.	1, 4
Banerjee et al. (2017), USA	To evaluate the learning of communication skills from a communication skills training program for nurses.	Acute care, pediatrics, critical care, and urgent care	Quasi-experimental	Inpatient oncology nurses (n = 342)	Clinician training: A 1-day program including lectures and roleplays with simulated patients.	Post-training survey on skills learnt and effectiveness of training. Retrospective pre/post survey on self-efficacy Pre/post assessment of roleplay with actor	>90% of participants indicated that they agreed or strongly agreed with five of the six evaluation items regarding perception of skills learnt >80% rated each individual module component as aiding in learning Nurses’ self-efficacy significantly improved Significant increase in overall skill use from pre- to post-training. The biggest gain was observed in empathic skills (other skill categories failed to demonstrate significance)	1, 4
Berns et al. (2017), USA	To assess whether a novel curriculum entitled Goals of Care Ambulatory Resident Education (GOCARE) improved resident physicians’ understanding of and preparedness for conducting ACP discussions in the outpatient setting.	Academic ambulatory care practice	Quasi-experimental	Second-year internal medicine residents (n = 42)	GOCARE clinician training: Small group sessions delivered over four weekly three-hour sessions. Included didactics, demonstration of skills, and roleplay.	Retrospective pre/post survey of residents’ self- rated attitudes, skills, and behaviors regarding outpatient ACP discussions. N = 29 completed 6 month follow up survey.	Statistically significant increase in residents’ self-rated preparedness for ACP discussions in both the inpatient and outpatient settings. Residents’ self-reported preparedness also was increased at the six-month follow-up.	1, 3
Casey et al. (2022b), USA	To determine the effect of an educational program for emergency physicians on ACP conversations in the ED during the COVID-19 pandemic.	ED In/out patient	Quasi-experimental	Adult patients with a confirmed diagnosis of COVID-19 in the 2 weeks up to and including the ED encounter of interest. (n = 143; n = 28 pre; n = 115 post) Clinicians (n = 72; 37 attendings and 35 residents)	ACP clinician training toolkit: A 60 minute virtual session including a guide to COVID-19 risk stratification, education on language to initiate ACP conversations, and modification of the electronic health record (EHR) to facilitate ACP documentation. System changes: templated “Smart Phrases” were created in the EHR to allow physicians to readily access the COVID-19 prognosis guide and to document ACP conversations.	pre-/post-descriptive statistics to measure occurrence of ACP activities	Observed a clinically meaningful increase in ED-based ACP activities in 25.4% of subjects across the pre- and post-intervention groups	3
Chatterjee et al. (2022), USA	To educate medical intensive care unit (MICU) clinicians, design and implement workflows relating to GOC communication, and measure the impact on communication proficiency and rate of GOC documentation	Medical intensive care unit	Quasi-experimental	Clinical fellows (n = 8)	3-Act Model clinician Training: three sessions over two weeks that included narrative reflection, online didactic and videos of 3-act model, and online pair roleplays	Post assessment of roleplays rated using Goals of Care Assessment Tool in online (roleplays) and in clinic (real patients) settings. Pre/post descriptive statistics to measure number of documented GOC conversations	All fellows achieved proficiency leading GOC conversation in at least one roleplay by the end of the training. All observed GOC discussions were rated as proficient. Documented GOC conversations significantly increased between pre-post periods	1, 3, 4
Day et al. (2022), Canada	To explore residents’ experiences with an online learning (e-learning) module that teaches a patient-centered approach to GoC conversations, and how the module might counteract the impact of the hidden curriculum on residents’ perceptions and approaches to GoC conversations.	IM at university hospital.	Qualitative	First-year residents (n = 11)	Clinician training: an electronic module that teaches a standardized patient-centered approach to GoC conversations.	semi- structured interviews	Themes identified from pre-module interviews: 1) the pressure to “get the DNR” shaped physician-centered approaches, and 2) emotional and moral distress resulted from pressures of the hidden curriculum. Themes identified from post-module interviews: 1) reconciliation of conflicting pressures, and 2) improved clinical encounters reinforced patient-centered approaches.	1, 3, 4
Doherty et al. (2023), Canada	To explore experiences of pediatric clinicians participating in a serious illness communication program (SICP) for advance care planning (ACP), examining how the SICP supports clinicians to improve their communication and the challenges of implementing new communication tools into clinical practice.	Two academic tertiary pediatric hospitals: the Children’s Hospital of Eastern Ontario (Ottawa) and British Columbia Children’s Hospital (Vancouver) in Canada in 2018.	Qualitative	Clinicians (n = 14)	SICP: a 2.5-hour in-person workshop that includes didactic presentations, demonstrations, role play scenarios, and a clinician guide.	Thematic analysis of interviews using interpretive description methodology.	Key themes included specific benefits of SICP, with subthemes of connecting with families, increased confidence in ACP discussions, providing tools to improve communication, and enhanced self-awareness and self-reflection. A second theme of perceived challenges emerged, which included subthemes of not having the conversation guide readily accessible, divergent team communication practices, and particular features of the clinical environment which limited the possibility of engaging in ACP discussions with parents.	
Epstein et al. (2017a), USA	To determine whether a combined intervention involving oncologists, patients with advanced cancer, and caregivers would promote patient-centered communication, and to estimate intervention effects on shared understanding, patient-physician relationships, QOL, and aggressive treatments in the last 30 days of life.	Community-based cancer clinics (4), academic medical centers (3) and community hospitals (3) in Western New York and Sacramento, California.	RCT	medical oncologists	Physician training: two sessions (1.75 hours) in-office training using a brief video, feedback from standardized patients Patient training: a one-hour patient and caregiver coaching session incorporating a question prompt list to help patients bring their most important concerns to their oncologist’s attention at an upcoming office visit, plus up to 3 follow-up phone calls	A composite of 4 prespecified communication measures matched to the goals of communication training (Active Patient Participation Coding [APPC]), responding to patients’ emotions (Verona VR-CoDES41), in- forming patients about prognosis and treatment choices (Prognostic and Treatment Choices [PTCC] Informing subscale) and balanced framing of decisions (PTCC Balanced Framing subscale) Intervention sessions were audio recorded and reviewed by lead trainers and investigators using a fidelity checklist.	the composite communication score showed a significant intervention effect (estimated adjusted intervention effect, 0.34; 95% CI, 0.06 − 0.62; P = .02) Of the individual communication component measures, only the engaging measure (APPC) was statistically significant. There were no statistically significant effects of the intervention on the PEPPI, THC, or HCCQ scales, or on 2-year survival and curability estimates; 2-year survival discordance was 59% in the intervention group vs 62% for control; corresponding figures for curability discordance were 39% and 44%. Fidelity was 94% or higher.	1, 4, 3
Fettig et al. (2022), USA	To examine the impact of a communication skills training workshop on coordination and collaboration among ICU team members.	ICU	Quasi-experimental	clinicians (nurses, physicians, social workers & chaplains; n = 36)	Clinician training: A communication skills training workshop based on VitalTalk that ran for 12hrs over 2 days, including presentations, discussions, and roleplays with actors simulating surrogates of critically ill patients.	Pre/post training surveys and 6 month follow up	Significant increase in RC (relational coordination) survey indexes from pre to 6 month post intervention. Participants rated their preparedness for various aspects of goals-of-care conversations after the workshop as higher in every area surveyed compared to before the workshop.	1, 5
Geerse et al. (2019), USA	To characterize the content of serious illness conversations and identify opportunities for improvement.	Outpatient oncology	Qualitative	N = 25 conversations from n = 16 clinicians (physicians, nurse practitioners, or physician assistants)	Serious Illness Conversation Guide. Clinicians received a 2.5-hour skills-based training to use the ‘‘Serious Illness Conversation Guide’’ (SICG).	Thematic analysis of interviews using interpretive description methodology.	The median conversation duration was 14 minutes (range 4 − 37), with clinicians speaking half of the time. Thematic analyses demonstrated five key themes: (1) supportive dialogue between patients and clinicians; (2) patients’ openness to discuss emotionally challenging topics; (3) patients’ willingness to articulate preferences regarding life- sustaining treatments; (4) clinicians’ difficulty in responding to emotional or ambiguous patient statements; and (5) challenges in discussing prognosis.	1, 2, 3
Haley et al. (2017b), USA	To determine whether brief education and electronic alerts increase the frequency of goals of care discussions.	Inpatient general medicine services at a tertiary care medical center	Quasi-experimental	N = 6 general medicine teams, with each team consisting of an attending physician, one resident, two interns, and a medical student.	SUPER Goals of Care Communication Tool for clinicians: Training included a 10 minute didactic presented during rounds on clinical units, laminated pocket card with the GOC communication tool. System changes: text alerts that were sent through the internal clinical messaging system to resident and attending physicians within 48 hours of admission if criteria was met. Included a link to the didactic tool and the specific criterion that prompted the alert.	Descriptive statistics on admission charts. Each chart was scored for 1) presence of a documented discussion of GOC any time after the alert, 2) change in code status after the alert, 3) noncode status limitations in care after the alert, 4) mention of hospice in the medical record after the alert, or 5) palliative care consultation	Significantly more patients had goals of care discussion documented in the medical record after tool was implemented, compared to the control period.	3
Kruser et al. (2017b), USA	To evaluate a structured training program designed to teach surgeons how to use BC/WC.	Acute care general, cardiothoracic, or vascular surgery at the University of Wisconsin	Mixed methods Quasi-experimental and Qualitative	Attending surgeons (n = 25)	The BC/WC tool: Clinician training was two hours, including didactics and roleplays with feedback. Within two months of training coaches met with surgeons individually to address questions and encourage utilization.	Quant: Scoring of transcripts of the final standardized patient conversations and each inpatient conversation to measure BC/WC Fidelity. Quant: Surveys (modified version of the Practitioner Opinion Survey) administered three and six months post training Qual: Open-ended face-to-face interview with patients and family.	Quant: Surgeons continued to achieve a median of 10 of 11 tool elements (range 7 − 11). Surgeons presented both a best and worst case for two distinct treatment options in 92% of conversations with hospitalized patients yet failed to make a clear treatment recommendation in 61% of conversations Quant: Three months after training, 96% of surgeons respondents reported that BC/WC is easy to use, 79% felt that the BC/WC tool is better than their usual approach for helping patients make decisions, and 71% reported actively using BC/WC in clinical practice outside the scope of the research study. However, only 38% of surgeons believed BC/WC saved time. Surgeon responses were sustained at six months. Qual: Patients and families found that BC/WC established expectations, provided clarity, and facilitated deliberation.	1, 2, 4
Lagrotteria et al. (2021), Canada	To explore clinicians’ experiences with the SICP 1 year after implementation.	General internal medicine wards of two Canadian teaching hospitals.	Qualitative	Clinicians (N = 23)	SICP: evidence- based questions and a conversation framework to explore, with topics including illness understanding, prognosis, values, goals, fears, sources of strength, essential abilities, acceptable tradeoffs, and family understanding. System change to identify at risk patients and template for documentation	Interviews were conducted individually using a semistructured guide and were 30 to 45 minutes in duration.	Three main themes: the ways in which elements of the SICP implementation (1) supported changes in clinician behavior, (2) shifted the focus of goals-of-care conversations with hospitalized patients beyond discussion of code status, and (3) influenced clinicians personally and professionally. (1) Changes in clinician behavior were supported by having a unit champion, interprofessional engagement, access to copies of the Serious Illness Conversation Guide, and documentation in the electronic medical record. (2) Elements of the program, especially the Serious Illness Conversation Guide, shifted the focus of goals-of-care conversations beyond discussion of code status and influenced clinicians on personal and professional levels. (3) Concerns with the program included finding time to have conversations, building transient relationships, and limiting conversation fluidity.	1, 3, 4
Moody et al. (2020), USA	To examine the effects of COMPLETE: a communication plan early through EOL to increase hospice enrollment in children with cancer at EOL.	Pediatric oncology	Quasi-experimental	Pediatric oncology fellow MDs (n = 3) and RNs (n = 3). Parents (n = 20) and children (n = 17). N = 13 followed to EOL, n = 3 still alive at last follow-up and n = 1 lost to follow up.	Clinician training of COMPLETE: a guided discussion of goals of care, using visual aids, with parents of children with cancer. Three day format based on VitalTalk.	Rate of hospice enrollment in children with cancer at EOL whose parents participated in Phase I or Phase II of the Communication Plan Early through End of Life (COMPLETE) pilot trials. PedsQL Cancer Module, Pain subscale parent-proxy form was used to assess pain in children using a Likert-type response for frequency of symptoms. The Parent Experience of Child Illness-Short Form Long-term Uncertainty subscale and Hearth Hope Index measured parents’ uncertainty and hope. Surveys for clinician self-assessment of preparedness for having goals-of-care discussions with parents, and course evaluation survey on usefulness Intervention fidelity and MD/RN empathy was assessed by two independent evaluators using a quality assurance checklist	Compared with previously published data, children of parents on COMPLETE had significantly higher rates of hospice enrolment. Compared with previously published data, children of parents on COMPLETE had significantly lower rates of high-intensity medical interventions at EOL. Parental hope and uncertainty; and child pain, although not significant, trended in favorable directions (n = 5 postintervention). Phase II COMPLETE training resulted in significantly improved self-assessed preparedness to engage in early goals-of-care discussions compared with baseline Clinicians reported that COMPLETE training was important or very important to the development of their own clinical skills. Five of six participants evaluated the quality of training as very good or excellent; one was neutral. All participants would recommend this training for pediatric oncology MD/RNs. Five of six participants thought it should be required for all fellows; one was neutral.	1, 2
Ouchi et al. (2023b), USA	To determine the feasibility of an emergency department-based, brief motivational interview to stimulate serious illness conversations among seriously ill older adults by trained nurses.	ED at one academic medical center and one community hospital	Quasi-experimental		ED GOAL: Training includes a one hour didactic on the research methodologies, motivational interviewing, and serious illness conversation skills, a four hour communication training with trained actors, bedside coaching by a doctorate-level, nurse champion with specialty-level certification in palliative care (SR) after every patient enrolment post-training.	Descriptive statistics of recruitment, intervention administration, and retention to measure feasibility. Advance Care Planning Engagement Survey (ACPES) to measure patient-reported readiness for serious illness conversations at baseline and follow up, and how well participants felt heard and understood about the medical care they would want if they were to get sicker. Proportion of participants who self-reported having spoken to their primary outpatient clinicians about their wishes for end-of-life medical care at follow-up. Changes in serious illness conversation documentation in the medical records including new healthcare proxy, medical order for life-sustaining treatment form, and conversations about goals of care for the following 6 months.	Recruitment: > 50% of eligible and willing patients are enrolled; Intervention administration: > 50% of enrolled patients complete the intervention with our trained nurses; and Retention: > 50% of enrolled patients can complete the outcome assessments. ACPES: The composite score increased from 3.63 to 3.72 out of 5 one month after the intervention (p = 0.38). 12 patients reported that they talked to their primary outpatient clinician about their future care 1 month after the intervention. 37 patients reported that they talked to their families about their future care preferences 1 month after the intervention. Most participants reported that they felt “completely” heard and understood about what they would want in medical care if they were to get sicker by the study nurse (n = 16 out of 26, 61.5%) compared to their outpatient clinicians (n = 4 out of 26, 15.4%) after our intervention A systematic review of the electronic medical records demonstrated that 16% (n = 12/76), 25% (n = 19/76), and 33% (n = 25/76) had new documentation of serious ill- ness conversations with their outpatient clinicians at 1, 3, and 6 months, respectively.	2, 3, 4
Pasricha et al. (2020), USA	To examine the feasibility, acceptability, and utility of a standardized serious illness conversation (SIC) to guide communication between nonpalliative care trained providers and surrogates of critically ill, mechanically ventilated patients.	Penn Presbyterian MICU in Philadelphia, Pennsylvania.	Mixed methods Quasi-experimental and Qualitative	Clinicians (n = 9) Surrogates (n = 19)	SIC guide to communicate with surrogates of mechanically ventilated patients within 48 hours of admission. Clinicians completed 3 hour training sessions, including roleplay with facilitators. System changes: An ACP note was created in the patients’ EHRs with prepopulated SIC questions for provider to ask and document during the in-person interview.	Quant: Surveys for surrogate evaluation of tool. Quant: Surveys for clinician evaluation of tool. Quant: Documentation of ACP and SICs Qual: Qualitative analysis of the SIC responses were categorized into themes	Quant: 95% of surrogates found the SIC to be mostly or extremely worthwhile Quant: All clinicians found SIC guide valuable to patient care and easy to administer. Quant: Of 72 eligible patients, advanced care planning documentation was completed in 50 patients, including 36 SICs, for an advance care planning completion rate of 69% and an SIC completion rate of 50%. Qual: Most commonly identified themes were friend/family (66.7%), independence (55.6%) and social interactions (47.2%).	1, 2, 3
Smith (2017), USA	To use an advance directive document as a guide to initiate communication about ACP for young adults with high-risk cancer in a simulated clinical setting.	Clinical Education and Evaluation Laboratory (CEEL) of the University of Maryland, Baltimore.	Quasi-experimental	Nurses (n = 18)	An ACP guide specifically for adolescents and young adults, such as Voicing My Choices (VMC). Clinician training included prereading on VMC, and a 50 minute simulation with feedback and debriefing.	Pre/post survey on nurses’ changes in attitudes and confidence, and satisfaction with the training.	Each measure of self-confidence in ACP significantly increased after the simulation, including comfort in ability to initiate ACP in the practice setting (t = j4.01, P G .0009), confidence in ability to discuss ACP (t = j2.49, P G .02), confidence in ability to discuss ACP with patients younger than 18 years (t = j3.42, P G .003), and an acknowledgement that they possess the skills needed to have an ACP discussion with their patients (t = 0.002, P G .002). There was an overwhelmingly positive response to this simulation activity. The participants felt that the simulation should be held routinely and that ACP should be a regular part of conversations with patients.	1, 4
Wolfe et al. (2016), USA	To present didactic materials and a workshop-style, case-based, longitudinal approach for teaching communication skills to learners in pediatrics.	This resource can guide longitudinal trainee curriculum or à la carte for singular educational experiences for pediatric settings	Text	NA	Diadictic instruction based on available literature Watch and critique video performances of the communication skills and then perform these skills in facilitated small-group role-play sessions. Periodic assessments based on the Accreditation Council for Graduate Medical Education (ACGME) milestones, to provide more practice and feedback to learners regarding their skills.	**Learner Assessment** Self-reflection - Reflective exercise worksheet (Appendix J) asks clinicians to describe a single personal experience in sharing life-altering information during their clinical encounters and to reflect on their skill in communication. Self-assessment - Learners are encouraged to use the self-assessment (Appendix G) to gauge their learning. The facilitator guide (Appendix H) contains sample discussion questions to encourage groups to consider the topics in the resource guide together. *ACGME Communication Milestones* - Professionalism - Interpersonal Communication Skills **Evaluation of the Curriculum** - After each didactic/ workshop, participants should be given an opportunity to evaluate the content and the speaker (Appendix L). - During their four personalized video session debriefs, residents are encouraged to give feedback on the curriculum as a whole.	Evaluation data revealed a significant improvement in self-efficacy among participants on four primary learning objectives related to sharing life-altering information in pediatrics (from previous studies)	4

### Themes

Four themes emerged from the data, *Delivery of training programs, Clinician outcomes, Patient outcomes,* and *System outcomes.* Subthemes were identified within each theme (see Supplementary File 1).

Theme one, *Delivery of training programs*, highlights a shift from didactic learning to simulated learning, to align with evidence of increased effectiveness of learning for participants, with Subtheme 1, *Simulated learning*, highlighting the use of simulated learning in the teaching of communication skills, Subtheme 2 describing *Virtual training,* Subtheme 3 exploring the *Challenges and barriers* to training and GOPC, while Subtheme 4 describes the role of training in *Filling the gaps*.

*Simulated learning* (Subtheme 1) was commonly reported across included studies. Sixteen studies utilized roleplaying between clinicians and simulated patients to teach and practice communication skills (Aaronson et al. 2023; Annadurai et al. 2021; Banerjee et al. 2017; Berns et al. 2017; Chatterjee et al. 2022; Doherty et al. 2023; Epstein et al. 2017; Fettig et al. 2022; Geerse et al. 2019; Kruser et al. 2017; Lagrotteria et al. 2021; Moody et al. 2020; Ouchi et al. 2023b; Pasricha et al. 2020; Smith 2017; Wolfe et al. 2016). Authors of the included studies advocated for utilization of simulated learning as it provides a realistic and safe environment to practice (Banerjee et al. 2017; Smith 2017; Wolfe et al. 2016), more opportunities to practice skills (Doherty et al. 2023), increases clinicians’ confidence (Doherty et al. 2023), encourages self-reflection (Berns et al. 2017; Doherty et al. 2023; Fettig et al. 2022), increased clinician’s understanding of patients’ perspectives (Aaronson et al. 2023; Doherty et al. 2023), and could result in greater learning and acquisition of communication skills (Banerjee et al. 2017; Berns et al. 2017; Doherty et al. 2023; Smith 2017; Wolfe et al. 2016). Roleplay scenarios were beneficial as they could be adapted to clinicians’ settings (Chatterjee et al. 2022; Fettig et al. 2022) and any areas of difficulty identified in prior to participants attending the workshops (Wolfe et al. 2016).

*Virtual training* (Subtheme 2) and online formats are emerging across GoPC communications skills training, with two studies utilizing completely virtual training formats (Casey et al. 2022; Day et al. 2022), and two (Chatterjee et al. 2022; Ouchi et al. 2023) using a combination of in-person and virtual methods. Virtual training included didactics (Casey et al. 2022; Chatterjee et al. 2022; Ouchi et al. 2023), demonstrations (Casey et al. 2022; Chatterjee et al. 2022; Ouchi et al. 2023), roleplay conversations (Chatterjee et al. 2022; Ouchi et al. 2023), and virtual assessment of communication skills (Chatterjee et al. 2022). Virtual delivery increased the feasibility of training programs and increased the reach of training programs (Ouchi et al. 2023) by providing workarounds for policies that restricted in person contact (Chatterjee et al. 2022; Ouchi et al. 2023), and provided participants flexibility, allowing them to participate around busy schedules, or not be restricted by distance (Ouchi et al. 2023). All studies that used virtual training methods found that virtual learning was effective in supporting clinicians to approach GoPC conversations (Chatterjee et al. 2022; Day et al. 2022; Ouchi et al. 2023).

Many *Challenges and barriers* (Subtheme 3) that impact training were identified by clinicians. Studies highlighted clinician’s competing priorities (Aaronson et al. 2023; Epstein et al. 2017), time constraints (Aaronson et al. 2023; Day et al. 2022; Doherty et al. 2023; Lagrotteria et al. 2021; Pasricha et al. 2020; Wolfe et al. 2016), as barriers to attending GoPC training. Studies also discuss challenges and barriers to implementing communication training or tools. These include training being resource intensive (Banerjee et al. 2017; Kruser et al. 2017), unfeasible training structures (Berns et al. 2017), having to prioritize clinical duties (Kruser et al. 2017), and difficulty adapting conversation guides into real conversations with patients (Lagrotteria et al. 2021).

GoPC communication training has the potential for *Filling the gaps* (Subtheme 4) in clinicians’ knowledge and skills. Studies report improvement in the uptake of the communication skills in areas where clinicians have traditionally received limited training, such as outpatient settings (Berns et al. 2017). Similarly on-the-job communication training for nursing staff (Doherty et al. 2023) and residents (Berns et al. 2017) who reportedly received less instruction and practice on communication as part of undergraduate and postgraduate training, prepares them to engage in GoPC conversations. Studies also highlighted how GoPC communication can improve serious illness conversations (Doherty et al. 2023), responding to difficult situations and questions (Doherty et al. 2023), and empathy skills (Banerjee et al. 2017).

Theme two, *Clinician outcomes*, explores how training programs impacted *Confidence with GoPC conversations* (Subtheme 1), S*kills regarding GoPC conversations* (Subtheme 2), *Clinician wellbeing* (Subtheme 3), and *Communication and collaboration* (Subtheme 4).

Clinician’s C*onfidence with GoPC conversations* (Subtheme 1) changes after receiving communication skills training. Eight studies have reported increases in clinicians’ confidence, or reductions in anxiety about having GoPC conversations (Annadurai et al. 2021; Banerjee et al. 2017; Berns et al. 2017; Doherty et al. 2023; Fettig et al. 2022; Moody et al. 2020; Pasricha et al. 2020; Smith 2017). This has included improved confidence in initiating conversations (Smith 2017), discussing prognosis (Annadurai et al. 2021), discussing treatment options (Berns et al. 2017; Fettig et al. 2022), expressing empathy (Banerjee et al. 2017; Fettig et al. 2022), discussing spiritual issues (Fettig et al. 2022), eliciting patient values, concerns and preferences (Annadurai et al. 2021; Banerjee et al. 2017; Berns et al. 2017; Fettig et al. 2022), and having difficult discussions(Banerjee et al. 2017; Berns et al. 2017).

Similarly to confidence*, Skills regarding GoPC conversations* (Subtheme 2), change in clinician’s skills after receiving communication skills training. Nine studies report an increase in utilization of communication skills (Aaronson et al. 2023; Annadurai et al. 2021; Banerjee et al. 2017; Berns et al. 2017; Chatterjee et al. 2022; Doherty et al. 2023; Geerse et al. 2019; Kruser et al. 2017; Moody et al. 2020), such as initiating conversations and engaging patients (Doherty et al. 2023; Geerse et al. 2019), exploring and clarifying patients’ understandings (Aaronson et al. 2023; Banerjee et al. 2017; Berns et al. 2017), exploring patient feelings, values, concerns and preferences (Aaronson et al. 2023; Annadurai et al. 2021; Geerse et al. 2019), disclosing concerns (Aaronson et al. 2023), responding empathetically (Banerjee et al. 2017; Berns et al. 2017), responding to emotions (Epstein et al. 2017), active listening (Doherty et al. 2023), engaged in reflective practice (Doherty et al. 2023), making engaging statements (Epstein et al. 2017), and discussing prognosis and treatment options(Aaronson et al. 2023; Epstein et al. 2017; Kruser et al. 2017). Five studies (Annadurai et al. 2021; Banerjee et al. 2017; Epstein et al. 2017; Geerse et al. 2019; Kruser et al. 2017) found no significant difference in some clinical skills before and after training, such as assessing patient/family understanding (Annadurai et al. 2021), discussing prognosis, particularly with specific timelines (Annadurai et al. 2021; Geerse et al. 2019), avoiding use of medical jargon (Annadurai et al. 2021), checking for understanding (Annadurai et al. 2021; Banerjee et al. 2017), providing a summary (Annadurai et al. 2021), empathy skills, such as acknowledging and validating patients’ feelings (Banerjee et al. 2017), and responding to emotions (Annadurai et al. 2021), questioning skills, such as asking open-ended questions, or encouraging questions (Banerjee et al. 2017), and agenda setting, such as information organization skills, or checking skills (Banerjee et al. 2017). One study found that utilization of skills depended on the physician, suggesting that underlying physician attributes and institutional norms may also impact skill utilization after training (Epstein et al. 2017).

Communication training can support, *Clinician wellbeing* (Subtheme 3). Three studies (Banerjee et al. 2017; Day et al. 2022; Lagrotteria et al. 2021) discuss how communication training can reduce moral distress (Banerjee et al. 2017; Day et al. 2022; Lagrotteria et al. 2021), bring meaning to work (Lagrotteria et al. 2021), improve clinicians’ satisfaction (Lagrotteria et al. 2021). Day et al. (2022) describe how after training, residents felt greater alignment with their ethical standards, and increased tolerance of uncertainty and complexity involved in GoPC decisions, which reduced emotional and moral distress, and alleviated the impact of perceived pressures around GoPC conversations.

Improved *Communication and collaboration* (Subtheme 4) are direct benefits of team-focused and interdisciplinary communication training. Six studies provided training to interprofessional teams, which included nurses, physicians, social workers, chaplains, medical doctors, and advanced care providers (Doherty et al. 2023; Fettig et al. 2022; Geerse et al. 2019; Moody et al. 2020; Pasricha et al. 2020; Wolfe et al. 2016). Training with a multidisciplinary focus fostered close partnerships between disciplines (Chatterjee et al. 2022; Doherty et al. 2023; Fettig et al. 2022), which resulted in an increase in palliative care consultations (Chatterjee et al. 2022), task integration across teams (Fettig et al. 2022), and improved communication between teams (Doherty et al. 2023). Collaboration within teams was facilitated by senior team members coaching junior staff (Banerjee et al. 2017), or by having experienced clinicians provide mentorship to teams in a champion role (Chatterjee et al. 2022; Lagrotteria et al. 2021; Ouchi et al. 2023).

Theme three, *Patient outcomes*, explores how training programs *Reduced uncertainty* (Subtheme 1), *Increased readiness for conversations* (Subtheme 2), facilitated *Communication between patient and practitioners* (Subtheme 3), and *Surrogate decision makers* (Subtheme 4), and promoted *Patient-centered care* (Subtheme 5).

Communication training for clinicians and/or patients, families and carers *Reduced uncertainty* (Subtheme 1) regarding patients’ prognoses and treatment options. Communication training facilitated reducing uncertainty without reducing hope (Moody et al. 2020), and increased patients’ understandings of what might happen in the future (Aaronson et al. 2023), with patients reporting that felt they were able to ask important questions (Epstein et al. 2017).

Communication training for patients and families *Increased readiness for conversations* (Subtheme 2), and their preparation to engage in goals of care conversations. Epstein et al. (2017) reported that patients who received communication training were more active partners in care, demonstrating greater assertiveness, asking questions, requesting clarification, expressing opinions and preferences to a greater degree more control patients. Communication training also increased participants’ self-reported readiness to talk to their clinicians about their goals for end-of-life care (Ouchi et al. 2023).

Similarly, communication training facilitates more effective *Communication between patient and practitioners* (Subtheme 3), and families. Communication training promoted improved communication between clinicians and patient/families (Banerjee et al. 2017; Day et al. 2022; Epstein et al. 2017; Pasricha et al. 2020; Wolfe et al. 2016), greater involvement of family members and other members of a patient’s healthcare team in goals of care discussions (Aaronson et al. 2023; Epstein et al. 2017; Kruser et al. 2017; Pasricha et al. 2020), closer relationships between clinicians and patients/caregivers (Chatterjee et al. 2022; Day et al. 2022; Pasricha et al. 2020), greater understanding of families’ emotions (Doherty et al. 2023). The training also provides effective structure to conversations (Doherty et al. 2023), to gather information (Pasricha et al. 2020) and greater alignment between patient, caregiver, and clinician expectations (Epstein et al. 2017; Pasricha et al. 2020).

Subtheme 4, addresses the importance of training emphasizing the role of *Surrogate decision makers*, in GoPC conversations (Berns et al. 2017). Involvement of surrogate decision makers after communication training (also referred to as Health Care Decision Makers) increases advanced care planning activities that are aligned with the patients’ values (Casey et al. 2022). Even when the patient died during hospitalization, surrogates reported that the conversation was worthwhile, as it increased their understanding of the patient’s condition and they felt a greater sense of control, aligned with a strong relationship with the patient’s care team (Pasricha et al. 2020).

Subtheme 5, *Patient-centered care*, explores how communication training affected patient care and outcomes. Studies reported greater understandings of patients’ goals, values and preferences after communication training, which allowed patients’ care to be better aligned with their wishes (Aaronson et al. 2023; Day et al. 2022; Doherty et al. 2023; Geerse et al. 2019; Ouchi et al. 2023; Pasricha et al. 2020), and greater provision and discussion of treatment choices (Kruser et al. 2017). Studies noted a shift from an emphasis on code status to patient-centered approaches to care that focused on patient values and shared decision making (Chatterjee et al. 2022; Day et al. 2022; Lagrotteria et al. 2021). One study found that children of parents involved in a communication program had significantly higher rates of hospice enrolment and lower rates of high-intensity medical interventions at end of life (Moody et al. 2020). In contrast, Epstein et al. (2017) found that communication training had no effect on quality of life or aggressive treatments and hospice use in the last 30 days of life. Another study noted that surrogates described their conversations as mostly or extremely worthwhile, even when patients died during hospitalization (Pasricha et al. 2020).

Theme four, *System outcomes*, explores how training programs impacted *Documentation of GoPC conversations* (Subtheme 1), and *Occurrence of GoPC conversations* (Subtheme 2).

Communication training positively influenced, *Documentation of GoPC conversations* (Subtheme 1). Studies reported increases in documentation of GOPC conversations (Casey et al. 2022; Chatterjee et al. 2022; Haley et al. 2017; Ouchi et al. 2023), which were supported by formalization of documentation procedures (Annadurai et al. 2021; Lagrotteria et al. 2021), changes to electronic medical records to allow documentation and retrieval (Berns et al. 2017; Casey et al. 2022), and having a documentation template in the electronic medical record (Aaronson et al. 2023; Casey et al. 2022; Pasricha et al. 2020).

Finally, communication training also influenced the *Occurrence of GoPC conversations* (Subtheme 2). Studies reported increases in rates of GOPC conversations (Ouchi et al. 2023), and documentation (Casey et al. 2022; Chatterjee et al. 2022; Haley et al. 2017; Ouchi et al. 2023), which was reportedly supported by implementation of electronic identification of patients that would benefit from GOPC discussion (Aaronson et al. 2023), and electronic reminders or cues from supporting clinicians (Lagrotteria et al. 2021) to ensure all patients received opportunities to have GOPC conversations when appropriate (Haley et al. 2017).

## Discussion

The aim of this integrative review of the literature was to evaluate and synthesize research that has investigated interventions to train registered health professionals to effectively communicate with patients in acute settings who are establishing their goals of care, to develop an understanding of current practices and their effectiveness. Effective communication in healthcare is essential for building trust, ensuring mutual understanding and shared decision-making (Simon et al. 2021).

Like literature review by Bakke et al. (2018) regarding communication training for health professionals, the studies included in this review highlight a growing trend towards interventions that utilize role-play to both teach and evaluate communication skills. Although the literature suggests many benefits to simulated learning, virtual training and other face-to-face pedagogies, it is evident that there are some persistent challenges and barriers to their widespread and consistent use and adoption. Clinicians report the difficulties they encounter when attempting to balance the demands of their clinical responsibilities with their desire and the patients’ need for thorough and empathetic communication (Kruser et al. 2017). Further complicating the barriers to accessing communication training, healthcare provides infrequently prioritize or fund this training to the extent that is required (Aaronson et al. 2023). As a result, patient care can be negatively impacted, their goals and values may not be adequately understood, documented or addressed, and patient and clinician satisfaction is compromised (Lagrotteria et al. 2021). Blended to approaches to communication training may overcome some of these barriers by providing accessible, high-fidelity learning opportunities for clinicians (Cappi et al. 2019).

The shift from didactic learning to simulated learning highlighted in the review aligns with the broader trends evident in the healthcare communication literature (Voogt et al. 2022) where there has been a consistent shift towards experiential and interactive approaches. The use of simulation and roleplaying across the included studies, also reflects the value of experiential learning in healthcare education (Martin et al. 2020), and its ability to bridge the gap between the theory and application of communication (Elendu et al. 2024). The included studies explored communication training for professionals working in a variety of settings, such as ED, ICU, outpatient settings etc., across a range of specialties (for example pediatrics, oncology).

Effective communication is complex, but communication training can improve clinician confidence and skill, and fill knowledge and skills gaps. Communication is a core clinical competency and integral to the delivery of patient-centered care (Sharkiya 2023). The finding that clinicians require the skills and knowledge to build relationships and facilitate complex decision making, in order support patients to plan their goals of care is consistent across disciplines (Bornman and Louw 2023). Communication training that adopts contemporary approaches to delivery, opportunities for practice and is contextually specific, can support clinician readiness for GoPC (Doherty et al. 2023), decrease their hesitancy (Smith 2017) and improve communication with their patients (Pasricha et al. 2020; Wolfe et al. 2016) and surrogates (Pasricha et al. 2020; Wolfe et al. 2016). Where clinicians have the skills to communicate effectively, patient’s wishes, goals and values are more likely to be addressed and met (Sharkiya 2023). An emphasis on clear and compassionate communication ensures that the emotional complexity is sensitively managed (Malenfant et al. 2022). A thorough approach to care that includes a comprehensive and documented GoPC can improve patient, carer and clinician satisfaction (Lagrotteria et al. 2021), especially where there is systemwide support and infrastructure in place (Berns et al. 2017; Casey et al. 2022).

Patient-centered care relies on effective team-based communication and collaboration (Dahlke et al. 2020), as well as effective clinician-patient communication (Santana et al. 2018). While a number of studies (Aaronson et al. 2023; Doherty et al. 2023; Fettig et al. 2022; Lagrotteria et al. 2021) provided training to a range of health professional roles, most studies tailored training towards nurses (Banerjee et al. 2017; Geerse et al. 2019; Moody et al. 2020; Smith 2017), or physicians (Annadurai et al. 2021; Berns et al. 2017; Casey et al. 2022; Chatterjee et al. 2022; Day et al. 2022; Epstein et al. 2017; Geerse et al. 2019; Haley et al. 2017; Kruser et al. 2017; Moody et al. 2020; Ouchi et al. 2023; Pasricha et al. 2020; Wolfe et al. 2016)).

The included multidisciplinary studies reflect a recent increase in GoPC communication training for specialists and allied health professionals (i.e. not just generalist doctors and nurses), which mirrors prioritization of multi-disciplinary care and greater collaboration within teams. Clinician GoPC-focused training the has the potential to increase the occurrence of conversations, while positively influencing how patients understand and can express their goals to the treating team (Kruser et al. 2017), to ensure their care is appropriately aligned to their wishes (Geerse et al. 2019; Ouchi et al. 2023).

Importantly GoPC training for patients and their surrogate decision makers was able to improve their readiness for engagement in GOPC conversations (Epstein et al. 2017; Ouchi et al. 2023). The widespread implementation of GoPC communication training is improved by healthcare and system processes, (Lakin et al. 2016), which improve the continuity of patient care, reduce errors and improve the overall quality of healthcare delivery (Haley et al. 2017). The implementation of systemwide initiatives such electronic medical records which prompt clinician action (Aaronson et al. 2023) can improve the uptake of GoPC training program outcomes, resulting in higher quality patient care and improved system efficiency.

In summary, both individuals and organizations, should prioritize communication training. Training should be regularly provided, context specific and promote the use of conversation guides. Individuals should be encouraged to embed GoPC conversations into their practice to promote effective and patient-centered communication. Formalizing the integration of communication skills training into pre-registration education and providing regular post-graduate professional development for clinicians should be carefully considered. Additionally, policies that address the barriers of time and resources, through the adequate allocation of funding and support should be developed and implemented. The adoption of technologies that facilitate timely GoPC will promote standardized and consistently applied approaches to GoPC. As practice and policy change, further research is required to understand the sustained effects of GoPC training on clinicians and patients, across clinical contexts. Assessment of the impact of various approaches to training that address the barriers and challenges identified in this review are warranted. Further research should also explore the role of GoPC training in improving patient-centered care, patient satisfaction, adherence to treatment and overall patient outcomes. Finally, further research should adopt measures beyond self-assessment and explore translation of knowledge and simulation-demonstrated skill into clinical settings.

While there are a number of useful implications for practice and policy these should be interpreted with caution as there was a lack of homogeneity in the approaches used to explore the effectiveness of communication training to improve GOPC conversations in acute care. There were also a number of methodological weaknesses across the studies including a lack of control groups (in quantitative studies) and statements locating the researchers culturally or theoretically (in qualitative studies).

## Conclusion

This review of the literature demonstrates that there is a shift from traditional didactic GoPC training towards experiential learning, supported by simulation and other practical opportunities. Approaches to training that adopt these strategies assist clinicians to develop communication skills that are nuanced to the complex environments in which they work. Despite the benefits of GoPC communication training, there are system barriers that preclude the effective and widespread implementation. Overcoming these barriers through strategies that resource and prioritize regular and timely training has the potential to ensure that clinicians can meet the GoPC needs of their patients. Effective, patient-centered communication builds trust, satisfaction and collaboration, constructs that are key to aligning patient care with their values and goals. In addition, there are clear benefits to the healthcare system through improvements in documentation and frequency of GoPC conversations, resulting in higher quality patient care.

## Supporting information

Brown and Hu-Collins supplementary material Brown and Hu-Collins supplementary material

## Supplementary material

The supplementary material for this article can be found at https://doi.org/10.1017/S1478951525000264.
