# Supplementary material for: Communication training for effective Goals of Patient Care conversations in acute care: An integrative review of the literature
Source: Palliat Support Care. 2025 Mar 28;23:e81. doi: 10.1017/S1478951525000264 (PMC13166658; doi:10.1017/S1478951525000264)
Supplement: Brown and Hu-Collins supplementary material [file S1478951525000264sup001.docx]

Themes

1. Delivery of training programs

- Simulated learning
- Virtual training
- Challenges and barriers
- Filling the gaps

1. Clinician outcomes

- Confidence with GOPC conversations
- Skills regarding GOPC conversations
- Clinician wellbeing
- Communication and collaboration

1. Patient outcomes

- Reduced uncertainty and increased readiness for conversations
- Communication between patient and practitioners
- Surrogate decision makers
- Patient-centred care

1. System outcomes

- Documentation of GOPC conversations
- Occurrence of GOPC conversations

|  | Simulated learning | Virtual training | Filling the gaps | Challenges and barriers | Confidence with GOPC conversations | Skills regarding GOPC conversations | Clinician wellbeing | Communication and collaboration | Reduced uncertainty  &  increased readiness for conversations | Communication between patient and practitioners | Surrogate decision makers | Patient-centred care | System outcomes |
| --- | --- | --- | --- | --- | --- | --- | --- | --- | --- | --- | --- | --- | --- |
| **(Aaronson et al., 2023)**  Assessing the acceptability and feasibility of leveraging emergency department social workers' advanced communication skills to assess elderly patients' goals and values | From qual focus group: “The training that we received felt rigorous with the actors.  And that I thought was really helpful  The woman who came in from a patient-family perspective  - hearing her perspective and what their conversations were looking like, I found really valuable” |  |  | Challenges related to the content around code status, the challenges of the ED environment, competing priorities and to the lack of clear follow-up  Of the 65 patients randomized to the intervention group, social workers were able to complete a SIC for 43 (66.1%) of them. In intervention patients for whom the conversation was not completed (22), the most frequent reasons included social worker work load in other areas of the ED (45%), the patient was discharged or admitted to the hospital before the social worker could arrive (20%), the patient refused the conversation (10%), or the patient was unable to participate secondary to acuity or confusion (10%).  From qual focus group “I do think that having more information or training around  code status and the nuances. even if we ourselves aren’t getting into that. just to have a knowledge base around it. would’ve been helpful for me”  In the social work focus group, the clinicians did identify  some key areas to help improve the intervention. These included a system to ensure that the conversations are seen, and continued, by the inpatient team or outpatient primary care physician (PCP). This has the potential to increase the efficacy of the intervention, and will require a solution that is likely both process based and technology enabled. The social workers also noted a need to identify how best to engage families. |  | Social workers consistently administered 95% of the intervention components. Within the conversation categories, assessment of prognostic awareness, exploration of what is important, and recommendations were complete 95% of the time. Disclosure of worry was completed 93% of the time. |  | Related to the ED providers, there was concern about pushback: “Those pushback moments stuck with me, more than the  positive reactions, which were more than the pushback I just don’t know that the information that we got from  patients were as respected or given as much as if[a physician] went to the provider and said, ‘‘I had this conversation’’”  Themes related to areas that represented opportunities for improvements in the program included the desire for a closed-loop system to communicate recommendations to the team, a way to integrate family into the discussions, scripted language that facilitates social workers building on conversations that have already been started with other members of their care team (e.g., oncologists or primary care physicians), and tools that could help patients continue these conversations with other caregivers:  “Any way that the work could be integrated into rounds conversations might be helpful I would also think I would have added a piece of communicating with the patient’s PCP Giving a card to the patient.that says, ‘‘I’ve had a serious  illness conversation, I’m ready to talk about my code status,’’ or something like that that they could then hand to a provider that they trusted” | High rates of acceptability of gocc by patients  Of patients who received the intervention (43) and had  completed follow-up assessments (38), 77% reported that they appreciated the social worker bringing up their personal goals for the future; 72% reported they appreciated the social worker asking about their fears and worries; and 81% reported they liked the way the conversation about their illness was set up. While not specifically measuring acceptability as the aforementioned questions from the QSIC tool do, the survey also revealed that 44% reported that the conversation would influence how they prepared for the future, and 48% reported that that discussing their illness with the social worker increased their understanding of what might happen in the future.  From the patient perspective, a theme of openness, and willingness to engage become apparent:  “[the patients] want us to come. Patients are really open to sharing and getting into deep  thought with us Patients were really receptive to social work coming in and  having these conversations” | a way to integrate family into the discussions,  and tools that could help patients continue these conversations with other caregivers:  I would also think I would have added a piece of communicating with the patient’s PCP Giving a card to the patient.that says, ‘‘I’ve had a serious illness conversation, I’m ready to talk about my code status,’’ or something like that that they could then hand to a provider that they trusted” |  | This model has the potential to offer a scalable model for identifying patients’ goals and values early in their hospitalization and as a result, allow patients’ care to be better aligned with their wishes.  From qual focus group “It’s a lot closer to what people think of when they think about  social work, really getting to sit with someone and understand how their upbringing and their beliefs and general views of life impact their views about what kind of treatment they want” | Related to ongoing integration of ED providers in identifying patients that would benefit from palliative care engagement, following this study the SQ has been hardwired in the electronic medical record (EMR) at the study site as a best practice alert on a subset of patients. |
| **(Annadurai et al., 2021)** Impact of a novel goals-of-care communication skills coaching intervention for practicing oncologists | The INT was an experiential learning model that used a two-hour role-play and four individualized coaching sessions to further practice specific SPIKES and NURSE components. These real-time joint sessions, conducted during oncologists’ clinic sessions with their actual patients, were condensed from the four-day VitalTalk model. |  |  | Offering ‘‘I wish’’ statements and providing prognosis were the least utilized skills both before and after INT (despite the former being practiced in our INT), possibly because of oncologist lack of experience in ‘‘I wish’’ statements or discomfort with delineating prognosis. | Our findings suggest that oncologists report greater comfort in core communication skills than observed in practice at baseline (e.g., in discussing prognosis and eliciting patient and family concerns). | All oncologists in both trial arms reported being comfortable in discussing treatment options at base- line. We believe that the low rates of utilization of related skills (eliciting values, avoiding jargon, and checking or assessing for understanding) in both groups before INT demonstrate the need for training courses despite this comfort level.  In the post-INT audiotaped visits, INT oncologists were significantly more likely to elicit patient values (55% vs. 0%; p = 0.01). the INT was found to be significant in its effect on oncologists’ ability to elicit patient values (b = 0.34; p = 0.04) but did not lead to a significant change in the use of other core skills ((1) assessing patient/family understanding, (2) giving information about current condition, (3) avoiding use of medical jargon, (4) responding to emotions, (5) checking for understanding, (6) providing a summary.  The INT did not significantly increase oncologist assess- ment of patient or family understanding, or their use of NURSE statements. |  |  |  |  |  |  | The SICP showed improvements when using a systems-based methodology with self-documentation of communication skills in the electronic medical record (EMR). |
| **(Banerjee et al., 2017)**  The implementation and evaluation of a communication skills training program for oncology nurses | An important aspect of our training is the focus on  role plays. Whereas didactics help orient participants to the training and provide evidence and best practices from literature, role plays provide experiential learning for participants and can result in sustained learning of communication skills. We used SPs for role plays that allowed us to re-create a realistic communication experience for the participants. |  | The largest gains were observed in empathy skills,  which is highly significant for oncology nursing. Offering a communication skills training program that addresses relevant communication challenges that oncology nurses face highlights the commitment of the institution to encourage and support communication between nurses and patients, therefore focusing on patient-centered care. | Resource intensive: For instance, using participants to role play, presenting the training in large groups, and using hand-held cameras for recording role play interactions and feedback can be accomplished with limited resources. | Significant improvement in self- efficacy regarding communicating with patients in various contexts: . Overall, nurses’ self-efficacy significantly improved [t(1016) = 31.17, p < .001] from pre(M = 3.31, SD = .88) to post-training (M =4.05, SD = .65). In particular, nurse participants’ selfefficacy in responding empathically to patients significantly increased [t(340) = 18.59, p < .001] from pre(M = 3.59, SD = .69) to post-training (M =4.22, SD = .56). Similarly, nurse participants’ self-efficacy in discussing death, dying, and end-of-life goals ofcare significantly increased [t(338) = 21.52, p < .001] from pre- (M= 3.03, SD = 1.02) to post-training (M=3.99, SD = .70). Finally, nurse participants’ self-efficacy in responding to challenging family interactions significantly increased [t(336) = 15.11, p < .001] from pre(M = 3.30, SD = .79) to post-training (M =3.93, SD = .64). | Significant improvement in 3/5 empathic skills (encourage expression of feelings, normalize, and praise patient efforts) from pre- to post-training (non significant improvement in Acknowledge, non significant reduction in Validate)  significant increase was observed in one out of five questioning skills (clarify), but not Ask open questions, Restate, Endorse question asking, or Invite questions.  No significant gains from pre- to posttraining were observed in any of the agenda setting skills, information organization skills, or checking skills. | Providing a communication skills training can be considered as an institutional resource to invest in uplifting the morale of their nurses. | This commitment was also strengthened through the use of facilitators from each nursing discipline facilitating participants from the same discipline, providing effective role models. |  | communication skills training for oncology nurses (as well as for other health providers) is an effective way of improving communication between nurse and patient/families |  | “Offering a communication skills training program that  addresses relevant communication challenges that oncology nurses face highlights the commitment of the institution to encourage and support communication training between nurses and patients, therefore focusing on patient-centered care” | No change in agenda setting |
| **(Berns et al., 2017)** Goals of care ambulatory resident education: Training residents in advance care planning conversations in the outpatient setting | Results suggests the importance of deliberate practice, use of reflection, and application in real clinical encounters in teaching communication skills that have lasting impact on learners. |  | Approximately 40%of residents reported prior communication skill training and 34% reported some form of palliative care training during medical school or residency. Only three residents reported engaging in five or more outpatient ACP discussions in the past year, discussions that could have included healthcare proxy, code status, and/or goals of care discussions.  The results of our study also highlight that IM residents felt more prepared to engage in ACP discussions in the inpatient setting compared to the outpatient setting and this persisted after GOCARE. This might be explained by the disproportionately larger percentage of time most residents spend on inpatient rotations where goals of care discussions seem more common and routine. This argues for the importance of ACP curricula to be focused on outpatient scenarios and ways to incorporate these skills into outpatient practices. Further- more, our findings illustrate that an outpatient-directed curriculum can impact not only outpatient preparedness for these discussions but also translate to improved confidence with conversations in the inpatient setting. | GOCARE provides an alternative model that can be compatible with standard residency training structures, as the structure is feasible compared to other multiday intensive retreats. | Residents had an increase in self-rated preparedness to discuss ACP in both inpatient and outpatient settings and used the communication steps involved both immediately post GOCARE and six months later. Post-GOCARE, residents reported higher overall preparedness for engaging in ACP discussions in the inpatient setting compared to the outpatient setting at baseline (5.23 vs 4.00 on a 7-point Likert scale), as well as after the completion of GOCARE (6.28 vs 5.20).  6-months post-GOCARE, residents reported that they were more prepared after GOCARE in using the ACP communication steps for inpatient and outpatient clinical settings and engaging in ACP discussions in the outpatient setting.  The greatest change was seen in the following ACP discussion steps: choosing an appropriate healthcare proxy, giving bad news, eliciting goals of care, and discussions of treatment options, including comfort care in the outpatient setting. | Residents also reported a statistically significant increase in the use of communication skills taught in the course such as ‘‘Ask-Tell-Ask’’ (clarifying patient understanding) and ‘‘NURSE’’ statements in clinical encounters |  |  |  |  | The course also emphasized the involvement of surrogate decision-makers in the discussions and communication with other medical providers involved in patients’ care. |  | Upon chart review immediately after the completion of GOCARE, 40 residents (95%) documented in the EMR at least one ACP visit with 10 of these residents (20%) engaging in more than the required number of visits (>2 visits).  The electronic medical record (EMR) was re- configured to facilitate ACP documentation and retrieval. |
| **(Casey et al., 2022)**  Advance care planning for emergency department patients with COVID-19 infection: An assessment of a physician training program |  | To educate physicians on the ACP toolkit, a 60-minute virtual educational session was held during a routine departmental meeting available to residents and attendings. |  |  |  |  |  |  |  |  | HCDM (health care decision maker): increase in ACP activities was largely driven by increases in patient identification of a HCDM 🡪 ensures that patients’ care is consistent with their values. |  | templated “Smart Phrases” were created to allow physicians to readily access the COVID-19 prognosis guide and to document ACP conversations  observed a clinically meaningful but non significant increase in ED-based ACP activities (identification of ahealthcare decision-maker [HCDM], an order for a code status, or a documented GOC conversation.) in 25.4% of subjects across the pre- and post-intervention groups. The difference was largely driven by increases in patient identification of a HCDM, but also included increased documentation ofcode status and GOC decision-making conversations |
| **(Chatterjee et al., 2022)**  Integrating narrative goals of care in the medical intensive care unit: Impact on educational and clinical outcomes | The 3-Act Model similarly uses didactics and roleplays but, instead of relying on checklists, focuses on a highly adaptive and memorable approach grounded in narrative medicine principles.  A team of collaborators, including a PCCM fellow and attending, internal medicine resident, and palliative care physician and nurse, developed the roleplay scenarios to reflect commonly encountered situations in the Hopkins Bayview MICU  Roleplays were done online in breakout rooms with clinicians playing both patient and physician. | Virtual roleplay  For the roleplays, fellows paired up in online breakout rooms and took turns playing patient and physician, with observation and immediate 360-degree feedback facilitated by a palliative care trainer  Virtual assessment  In the clinical setting, trainers collected 14 unique GCAT ratings via observation of all fellows (8/8) leading GOC conversations with MICU patients. Given the restrictive visitor policies in place, most of these GOC conversations were conducted via a Health Insurance Portability and Accountability Act–compliant video platform. Typically, the medical team members (PCCM fellow, palliative care physician, and occasionally other MICU or palliative care clinicians) would huddle on video before admitting the family from the virtual waiting room. |  |  |  | All fellows who underwent the 3-Act Model training were found by objective assessment to be proficient in GOC conversation (both in role- play and with real patients)  Roleplay:  All fellows (8/8) achieved proficiency leading GOC conversation in at least one roleplay by the end of the training. Seventy-five percent (6/8) were proficient after one roleplay; the remaining 25% were rated proficient by their second roleplay. In total, 71% (17/24) of all roleplay attempts were rated proficient by the trainers.  Clinical setting:  Every fellow achieved proficiency in leading at least one GOC conversation. All observed GOC discussions (14/14) were rated as proficient. Half of the fellows (4/8) received ratings on two GOC discussions; the remainder received ratings on either one (3/8) or three (1/8). Of fellows with at least two observed discussions (5/8), 40% improved in at least one domain of the GCAT between discussions.  . |  | Our interteam collaboration, guided by Lean Six Sigma principles, was intentionally interprofessional, leveraging preexisting ICU culture and resources. Through the educational sessions and collaborations in the hospital, the palliative care trainers formed strong rapport with the PCCM fellows. The culminative result was a close partnership, as exemplified by the dramatic increase in palliative care consults postintervention.  Lean Six Sigma methodology relies on a collaborative team effort to improve performance by reducing waste and variation. Key steps include defining the problem and its root cause, engaging stakeholders, designing system interventions, evaluating outcomes, and making iterative adjustments to the model as needed (16). The team determined that targeting the PCCM clinical fellows for GOC communication skills training and empowering them as leaders on the MICU team ofGOC communication would be a high-yield multicomponent intervention (Figure 1). We also proactively engaged PCCM fellowship program leadership, who recognized such training as a valuable and needed entrustable professional activity for the fellows (17).  through multidisciplinary rounds and bedside mentorship, the champions encouraged and empowered nurses and other members of the multidisciplinary team to pass on GOC insights about specific patients to the fellow.  Before each 4-week PCCM fellow rotation, the Medical Director (S.C.) discussed the goals and objectives of the ICU rotation, emphasizing leading GOC conversations and engaging the multidisciplinary team in support of the MICU holistic care model. Nurse rounding templates were also updated during the intervention period and included a prompt for code status, last update to family, family issues, and social work needs.  during monthly ICU faculty and nursing staff meetings during the intervention, the MICU Director explained fellows should serve as clinical lead for GOC communication and directed nurse staff to freely communicate perceived palliative care needs to the team, including during bedside rounds. Palliative care team members connected with PCCM fellows early in their rotations to give support and encourage collaboration. As positive reinforcement, palliative care consult physicians also celebrated PCCM fellows who demonstrated excellence in leading a GOC conversation via email communication with the MICU Medical Director and the PCCM Program Director.  MICU nursing leadership identified several experienced and motivated nurses to serve as unit champions, with whom palliative care 3-Act Model leadership met serially to identify barriers and facilitators to effective GOC discussions. |  | Rather than reliance on check- lists and triggers, our intervention hinged on relationship building between clinicians and patients and their families, as well as between the palliative care and MICU teams. |  | Demonstrated objective skills proficiency in pulmonary and critical care medicine (PCCM) physicians resulting from a narrative approach to GOC communication training.  We believe  an ICU culture that prioritizes narrative and personhood is worth systematic pursuit. | Significant increase in documented CoPC conversations 5.55% (2/36) of patients during the preintervention period, and for 28.89% (13/45) in post.  Patients with a week-long MICU stay had a five- fold increase in the likelihood of having documented high-quality GOC conversations after intervention |
| **(Day et al., 2022)** "Get the DNR": Residents' perceptions of goals of care conversations before and after an e-learning module |  | (elearning) module to teach trainees a standardized, patient centered approach to leading GoC conversations with inpatients. The module is interactive and uses several evidence-based educational strategies, including the use of fictional patient cases, frequent reflective questions and exercises, and video role modeling of each stage of the approach to patient-centered GoC communication.  There is explicit teaching and labeling of effective communication strategies, such as the use of open-ended questions to solicit patient values. |  | In the pre-module interviews, while residents recognized that the intent of these conversations should ideally be to “[get] a better sense of the [patient’s] overall philosophy of care” (P5), they described multiple institutional and social pressures leading to physician-centered GoC conversations focused on code status (i.e., patient directives for cardiopulmonary resuscitation), documentation, and efficiency.  **Institutional pressure**: Hospital medico-legal requirements to document a code status at the time of patient admission encouraged conversations focused on code status. Needing to “check a box [on a form]” (P3), residents described presenting resuscitation choices like a “menu of options” (P9)  While residents felt conflicted that patients could be “rushed” (P11) into making potentially uninformed care decisions, they felt “forced” (P2) to comply with institutional requirements and prioritized paternalistic agendas  **Hierarchical pressures**: Resident descriptions of GoC conversations were further shaped by perceived hierarchical “expectations” (P11) not only to focus on code status, but explicitly to “get the DNR” (Do-Not-Resuscitate) (P4) even if it might be contradictory to patients’ beliefs or values  Pressure to “get the DNR” (P7) was often conveyed explicitly by supervisors, as residents felt “pressured to have these discussions in relatively short periods of time when in reality [it] might take weeks” (P6). Sometimes, “pressure” (P1) was implied from negative role modelling, including depersonalized comments and modelled poor communication with families. At other times it was conveyed through repeated questioning or requests from a supervisor with an implied hidden agenda. | GoC conversations are sources of significant anxiety and apprehension, indicating that this distress may in part be fueled by the emotional dissonance that results from the contradiction between what is taught (formal curriculum) and what is modeled (hidden curriculum). |  | In the pre-module interviews, identified theme: 2) emotional and moral distress resulted from pressures of the hidden curriculum.  the post-module interviews found two additional themes: 1) reconciliation of conflicting pressures  emotional and moral distress resulted from  pressures of the hidden curriculum Moral distress was evident as trainees described, feeling “tugged at opposite ends” (P8) trying to manage perceived pressures to “get the DNR” (P4) with the ethical responsibility to respect patients’ autonomy and thus to do what is “right for the patient” (P4)  On one hand, they grappled with “guilt” (P8), feeling they might have pushed families into making decisions that they did not “fully comprehend” (P2). On the other hand, if they failed to get the desired code status, they felt as though they had disappointed their supervisor or colleagues  reconciliation of conflicting pressures After completing the module, residents described that they had developed new conceptualizations of and approaches to GoC conversations, in which they emphasized the importance of patient-centered over “paternalistic” (P11) priorities. Many reframed their definition of success in these conversations to be no longer contingent upon achieving the desired code status. This helped to reconcile conflicting tensions between formal and hidden curricula. Reflecting on prior “misconceptions” (P2) and attitudes  As a result, residents felt increasingly “confident” (P1) and empowered to overcome pressures from the hidden curriculum including institutional and social pressures that conflicted with the patient-centered approach. This change in mindset was driven by the following two subthemes: greater alignment with their ethical standards, and increased tolerance of uncertainty and complexity involved in GoC decisions.  Tolerance of uncertainty and complexity: After reflecting on the module content and applying the new strategies in the clinical setting, residents described increased comfort with patient uncertainty around GoC decisions. They described that the lessons from the module helped to alleviate the impact of perceived pressures to ensure patients had definitive GoC decisions made and documented at the time of admission. Reflecting on a recent patient experience, P6 commented that she no longer felt the need to focus primarily on code status documentation and efficiency in these conversations: |  |  | By promoting new patient-centered conceptualizations and attitudes in conversations that are often associated with poor communication and patient harm, this module contributed to improved quality of communication.  Improved clinical encounters reinforced patient-centered approaches When enacting their new approaches in the workplace, residents perceived that there was greater receptivity, “therapeutic alignment” (P7), relationship-building and “trust” (P2) with patients, in contrast to prior conversations that were frequently strained. Many residents described conversations with reduced tension, improved rapport, and better communication that, at times, led to unexpected praise from families. These reactions were especially transformative for those who were initially skeptical of the new approach and helped to affirm their commitment to new patient-centered attitudes and communication strategies. |  | patient narratives and role-modeling in the module may have served to promote a more holistic, relationship-centered approach to care, underpinned by empathy and shared-decision making.  In the pre-module interviews, we identified two themes: 1) the pressure to “get the DNR” shaped physician-centered approaches  In the post-module interviews we found 2) improved clinical encounters reinforced patient-centered approaches.  Greater alignment with internalized ethical standards: Residents described that the module’s role modeling of and emphasis on patient autonomy and shared decision aligned with their sense of professionalism and ethics compared to prior approaches they had adopted. This helped to shape new perceptions and expectations for GoC conversations that transcended pressures of the hidden curriculum  Residents described still documenting a code status designation, but now focused on broader discussions around patient values and wishes, recognizing that documented code status and treatment preferences could change with time and be modified accordingly. | we found that residents’ experiences were heavily influenced  by institutional policy and academic hierarchies, leading to the prioritization of physician-driven agendas and a task- oriented mindset at the expense of meaningful conversations around GoC. |
| **(Doherty et al., 2023)** | Participants noted that role play scenarios during the training workshops also enhanced their understanding of parents’ perspectives  Participants were exposed to a variety of clinical scenarios where they were able to role play the serious illness conversation, participants noted that this practice increased their confidence, noting that the role play “really helps to consolidate the knowledge and to practice” (P4). Participants noted that roleplaying SIC increased their confidence  Participants identified that the workshop role play was particularly important in allowing them to practice using the communication tools they had learned from the workshop.  Additionally, participants noted that observing the role plays also provided an opportunity for self-reflection |  | Clinicians identified that the workshop helped them know how to initiate serious illness conversations, identifying that this had previously been a barrier in ACP communication.  Participants identified challenging events in their clinical practice when the SICP had been particularly helpful, describing how specific phrases from the SICG guide helped them to respond to parents’ questions or during difficult moments in the conversations. | Participants described several challenges to implementing their learning, which occurred under several subthemes: guide accessibility,  - Not having a copy of the SICG with them at all times  divergent communication practices within the team,  - Different approaches to SIC amongst medical team  and clinical environment.  - Finding an appropriate setting for having a SIC. participants noted that the in-patient setting was particularly challenging due to the focus on acute issues and discharging the patient home as quickly as possible. Participants also describe feeling that they did have adequate time in their clinical workflow to have a serious illness conversation. | Participants felt more confident having serious illness conversa- tions | Participants felt the training improved their active listening skills which gave families more opportunities to share their feel- ings. Participants also identified how listening helped them to identify non-verbal cues which suggested that parents were not ready to have a detailed ACP discussion.  Participants noted how the guide became a tool for personal reflec- tive practice. Clinicians noted how they developed insights into their own communication practices and the limitations in their personal communication skills, which led them to develop tech- niques to improve their own communication.  Clinicians identified that the workshop helped them know how  to initiate serious illness conversations, identifying that this had previously been a barrier in ACP communication. Participants identified challenging events in their clinical practice when the SICPhad been particularlyhelpful, describing howspecific phrases from the SICG guide helped them to respond to parents’ questions or during difficult moments in the conversations. |  | participants who worked with team members familiar with the SICG noticed that there was improved conversation structure and overall ACP communication within their team. |  | After participating in the workshop, participants noticed they were better able to understand and connect with families’ emotions. Participants noted that they had started to realize that there is not a universal set of emotions to expect from families during serious illness conversations. Participants reported being able to better identify parental reactions including anxiety, anger, disbelief, or no reaction at all.  Many participants felt the SICG and workshop provided communication tools which helped them structure their communication with parents more effectively. |  | The workshop taught clinicians the importance of focusing on parents’ goals, worries, and values. Participants found that the SICP training provided them with tools for inquiring about and then listening to parents’ emotions and this helped clinicians to connect more deeply with families. |  |
| **(Epstein et al., 2017)** Effect of a patient-centered communication intervention on oncologist-patient communication, quality of life, and health care utilization in advanced cancer: The VOICE randomized clinical trial | The experimental intervention36 included (1) a 2-session in-office physician training (1.75 hours) using a brief video, feed- back from standardized patients portraying roles of patients with advanced cancer who also critiqued up to 2 audio recorded study patient visits, |  |  | “The current productivity-oriented practice environment also presents barriers to effective communication.” |  | As expected, utilization outcomes clustered by physician, suggesting that addressing underlying physician attributes and institutional norms might also be needed to address utilization of aggressive interventions and hospice.  The intervention resulted in clinically and statistically significant improvements in the primary physician-patient communication end point  The sample standard deviation of the composite from the prerandomiza- tion cohort was 0.53, hence the estimated intervention effect of 0.34 corresponds to a standardized effect of 0.64, corresponding to 5.7 additional “engaging” statements (a 44% increase), 0.6 additional responses to emotion (a 71% increase), and 1.4 additional statements regarding prognosis and treatment choices (a 38% increase).  clinically meaningful increases in responding to emotions and discussions of prognosis and treatment choices. |  |  | Single 1-hour patient and caregiver coaching session incorporating a question prompt list to help patients bring their most important concerns to their oncologist’s attention at an upcoming office visit, plusupto3follow-up phone calls  clinically meaningful increases in engaging patients in discussions  provision of information or emotional support, for example, may depend on a patient cue or request as well as a clinician’s willingness and capacity to respond.  Of the 4 communication domains, the most fundamental, engaging patients as active partners in care—being assertive, asking questions, requesting clarification, expressing opinions and preferences to a greater degree than control patients—was independently significant in secondary analyses. | an intentionally brief communication intervention was effective in improving patient-centered communication in advanced cancer  intervention focused on aligning patient, caregiver, and physician expectations.  Intervention did not affect Secondary outcomes ( the patient-physician relationship, shared understanding of prognosis, QOL, and aggressive treatments and hospice use in the last 30 days of life).  87% of patients “would recommend coaching to other patients with cancer”; and 85% were able to ask “all” or “most” of their “most important” questions. |  | approach was individualized and tailored to participants’ educational needs as identified in pre-questionnaire and interview |  |
| **(Fettig et al., 2022)**  A communication skills training workshop to improve ICU team relational coordination about goals of care: A pilot study | The training spanned 12 hours over two days and consisted of large group presentations and discussions led by course physician facilitators and communication skills practice in small groups with actors trained to simulate surrogates of critically ill patients. For simulation practice, participants were divided into two  small interprofessional groups with no more than six participants and one workshop facilitator. Each small group encountered three actors who portrayed family members of critically ill patients. Participants had an opportunity to volunteer to talk to the simulated family member one on one while the rest of the group observed. Each participant maintained their discipline role during the simulation. Facilitators made minor adjustments to the clinical scenario to reflect how participants from various disciplines might realistically encounter the family member in clinical practice. The facilitator used a reflective teaching practice to give each participant the opportunity to reflect on their own communication practice and how they might approach challenges in communication. |  |  |  | Participants rated their preparedness for various aspects of goals-of-care conversations after the workshop as higher in every area surveyed (eliciting family concerns at patient’s end of life, discussing religious/spiritual issues with families, responding to families who want treatment that is not indicated, discussing discontinuation of treatment, respoding to families who’s expectations do not match prognosis, discussing various treatment options, expressing empathy, doucting/participating in family meetings, discussing bad news) compared to before the workshop. (P <  .0001 |  |  | Team-based communication skills training may improve ICU team relational coordination surrounding goals of care. Seventeen and fifteen participants completed the relational coordination survey and JSAPNC respectively at both time points. The mean intergroup relational coordination index increased from 2.94 pre-intervention to 3.19 6-month post (P = .002, d= .89). Nurse relational coordination index ratings of all other groups increased from 2.84 pre-intervention to 3.08 6-month post (p = .004, d=1.23). Mean total scores on the JSAPNC survey did not change significantly from pre-intervention (53.8) to 6-month post (54.2, P =.45).  Relational coordination (RC) is defined as a “mutually reinforcing process of interaction between communication and relationships carried out for the purpose of task integration.”  after participating in an interprofessional communication training workshop about goals-of-care conversations, clinicians’ ratings of goals of care RC among team members improved significantly.  - Understand others’ skills  - Role sharing  - Increase in conversations about goc  - Shared challenges  Mutual respect among team members may have been enhanced directly by the workshop curriculum through greater role clarity and understanding ofareas ofoverlap, as well as a greater understanding of shared challenges and the emotional nature of the work for all interprofessional team members.  Additional workshop sessions were devoted to enhancing understanding the roles of each discipline in goals-of-care conversations as well as intrateam communication about conflicts in goals of care. In addition, the interprofessional composition of the small groups reinforced the concepts of role sharing in communication tasks and clarified areas of role divergence. |  |  |  |  |  |
| **(Geerse et al., 2019)**  A qualitative study of serious illness conversations in patients with advanced cancer | The skills-based training included additional information, a role playing exercise, |  |  | clinicians still experienced challenges in sharing prognostic information aligned with patient preferences, addressing emotions, clarifying concerns and preferences, and in following up on treatment limitations. | we also demonstrated that clinicians, despite (or be- cause of) their warm and comfortable relationships with their patients, frequently did not meet this standard, especially when discussing prognosis. Emotional discomfort on part of both the clinician and patient is likely to contribute to this pattern. Moreover, clinicians sporadically followed up with patients about their expressed preferences regarding life- sustaining treatments. Discussing the future with seriously ill patients can be an emotional experience, and often elicits anxiety, sadness, and fear | We found that training and a systematic framework allows clinicians to engage in these challenging conversations, provides space for patients to ex- press their thoughts and feelings, and explores basic values and QoL-issues while engaging patients in planning for the future.  we observed a prognostic discussion in ap- proximately half of the conversations and few clinicians provided patients with a time-based estimate (12%) even though the skills-based training suggested that clinicians tailor their prognostic disclosure to individual patient preferences. Instead, clinicians primarily focused on treatment options or discussed prognosis indirectly. |  |  |  |  |  | Clinicians in our sample provided space for patient-centered conversation and focused on what was important to patients, as supported by the finding that clinicians spoke approximately half of the time. |  |
| **(Haley et al., 2017)** Electronic goals of care alerts: An innovative strategy to promote primary palliative care |  |  |  |  |  |  |  |  |  |  |  |  | brief goals of care education and an electronic alert significantly increased the proportion of high-risk hospitalized patients with a documented discussion about goals of care.  Our study protocol did not, however, increase down- stream outcomes such as changes in code status or other limitations in care.  Electronic reminders are increasingly used to promote clinical pathways and standardize care. These systems can also be used to increase  communication about goals of care to ensure that every patient receives an opportunity to discuss their values and preferences. |
| **(Kruser et al., 2017)**  "Best case/worst case": Training surgeons to use a novel communication tool for high-risk acute surgical problems | each surgeon practiced using the BC/WC tool with two standardized patients in two different specialty- specific simulated cases | To address these barriers, we have developed an instructional video (https://www.youtube. com/watch?v1⁄4FnS3K44sbu0) linked to a training pro- gram to improve the scalability of this intervention |  | It is notable that surgeons reported discomfort providing a specific treatment recommen- dation as it conflicted with their understanding about how to support patient autonomy. This was reflected in their clinical use of the tool as less than half provided a treatment recommendation for hospitalized patients.  We relied on local experts to conduct the didactic sessions and one-on-one coaching, which is resource intensive. In addition, surgeons canceled and rescheduled multiple training and coaching sessions due to patient care and operating room conflicts, highlighting the burdens of a structured training curriculum for busy clinicians. To address these barriers, we have developed an instructional video (https://www.youtube. com/watch?v1⁄4FnS3K44sbu0) linked to a training program to improve the scalability of this intervention | 96% of surgeons respondents reported that BC/WC is easy to use, 79% felt that the BC/WC tool is better than their usual approach for helping patients make decisions, and 71% reported actively using BC/WC in clinical practice outside the scope of the research study. However, only 38% of surgeons believed BC/WC saved time | Although some surgeons easily incorporated patient preferences into treatment recommendations, our two-hour training session was not able to overcome deep-rooted cultural notions that many attending surgeons have about autonomy and surgical decision making  First, patients and families reported that surgeons who used BC/WC clearly defined treat- ment choices and encouraged comparison between the two treatments. Second, by showing the range of possible outcomes, surgeons established expectations and helped patients and families prepare for possible adverse events. Finally, patients and families valued the graphic aid as a tangible reference to facilitate future deliberation and inform family members who were not present for the discussion. Several patients and family members had saved the graphic aid months after hospitalization and reproduced the diagram at the follow-up interview without prompting.  It is notable that surgeons reported discomfort providing a specific treatment recommen- dation as it conflicted with their understanding about how to support patient autonomy. This was reflected in their clinical use of the tool as less than half provided a treatment recommendation for hospitalized patients.  When using BC/ WC in clinical practice with hospitalized older adults, trained surgeons continued to achieve a median of 10 of 11 tool elements (range 7e11). Surgeons presented both a best and worst case for two distinct treatment op- tions in 92% of conversations with hospitalized patients yet failed to make a clear treatment recommendation in 61% of conversations |  |  | First, patients and families reported that surgeons who used BC/WC clearly defined treatment choices and encouraged comparison between the two treatments. Second, by showing the range of possible outcomes, surgeons established expectations and helped patients and families prepare for possible adverse events. Finally, patients and families valued the graphic aid as a tangible reference to facilitate future deliberation and inform family members who were not present for the discussion. | Patients and families in our study used BC/ WC to visualize and prepare for treatment outcomes, suggesting their communication needs were met by the BC/WC strategy. |  |  |  |
| **(Lagrotteria et al., 2021)**  Clinicians' perspectives after implementation of the serious illness care program: A qualitative study | From study referenced that outlines intervention: The training begins with a brief didactic session (<30 min) on the evidence base for ACP discussions. A demonstration and discussion of the use of the SICG (approximately 30 min) are followed by individualised practice by all participants, using role-plays with personalised feedback from attending palliative care physicians (more than 60 min). Training sessions include 6–10 participants per session. Trained actors serve as standardised patients for both the demonstration and role-play. |  |  | Many described the presence of a unit champion as one of the most important program elements. One clinician said, “There’s a cueing reminder, there’s an administrative burden removed where someone else is scheduling and letting me know when it [the conversation] is, based on my availability, and that really helps. The patients and family are primed on it. It was just any time I wanted to do it, and whenever we planned to do it, it just happened. Everyone was on board, and everything was set up.” Many clinicians expressed opinions that were similar to 1 clinician’s statement that “the biggest challenge is sort of creating a formal time or space to have these conversations.” However, the unit champion’s coordination of a scheduled time to meet helped in overcoming this challenge: “I think the nudging from...[the unit] champion, supporting the program, is probably the thing that’s facilitated the most in terms of actually using the guide, of getting over that hurdle to make the time to do it.”  The inclusion of a formal documentation step within the program was described as bringing more attention to the conversation among the medical team and facilitating care over time that was more consistent with patient wishes.  When asked about which elements of the program facilitated participation, many clinicians referred to having copies of the guide accessible.  Although many clinicians stated that their comfort with conversations about serious illness increased because of the SICP, a few said that it could disrupt the natural flow of a conversation. One clinician said, “I just think just the static nature of it makes it a bit difficult to adapt to an actual, real- life conversation sometimes.”  Concerns with the program included finding time to have conversations, building transient relationships, and limiting conversation fluidity. |  |  | the SICP’s role in bringing meaning to clinicians’ work and reducing moral distress supports the fourth facet of the quadruple aim of health care, which is improving clinician satisfaction. |  |  |  |  | shifting the focus of goals-of-care conversations from an emphasis on code status to patient values, and influencing clinicians on personal and professional levels. | the multifaceted design of the SICP, especially the system- change components (the presence of a unit champion, the accessibility of the guide within units, and the formalized documentation procedure), supported behavior change among clinicians.  clinicians perceived changes in practice behaviors, suggesting that the system changes introduced by the SICP had benefits for workflow, including the cueing of practitioners and the creation of time and space for conversations  This finding emphasizes the importance of system change for successful implementation of the SICP; other studies have found that serious illness communication training alone was not associated with such behavior changes. |
| **(Moody et al., 2020)**  A pilot study of the effects of COMPLETE: A communication plan early through end of life, on end-of-life outcomes in children with cancer | MD/RN dyads were trained to deliver COMPLETE using didactic methods and role play with bereaved parents  Based on clinician feedback, Phase II training was revised to incorporate empathic communication skills and role play with actors. |  |  |  | - Phase II COMPLETE training resulted in significantly improved self-assessed preparedness to engage in early goals-of-care discussions compared with baseline | Our study is also consistent with published data showing that training oncology clinicians to use a guide for these discussions can result in high-quality EOL conversations. |  |  | children of parents on COMPLETE had significantly higher rates of hospice enrolment and lower rates of high-intensity medical interventions at EOL.  These discussions, which incorporate visual aids, result in high-quality EOL care and reduced parent uncertainty, without taking away hope. |  |  |  |  |
| **(Ouchi et al., 2023)**  An emergency department nurse led intervention to facilitate serious illness conversations among seriously ill older adults: A feasibility study | 1-h didactic on the research methodologies, motivational interviewing, and serious illness conversation skills followed by a 4-h communication training with trained actors | The COVID pandemic necessitated us to deliver ED GOAL virtually. the virtual delivery of ED GOAL seemed to increase its feasibility by: (1) allowing participants to conduct the interview at comfortable home locations while leveraging the clinical significance of their recent emergency department visits; and (2) centralizing the trained study nurses at our institution and potentially expanding the reach of ED GOAL in other EDs nationally  Due to COVID-19 restrictions prohibiting our research staff from being pre- sent in the emergency department, most participants were enrolled virtually after their emergency department visit using institution-approved Zoom.  Our trained study nurses delivered ED GOAL within 27 min virtually to all participants. 100% of enrolled subjects completed the intervention. Given most interventions were scheduled after the emergency department visit at the time of participants’ convenience and conducted virtually, none were inter- rupted. |  |  |  |  |  |  | ED GOAL led to significant increases in participants’ self-reported readiness to talk to their outpatient clinicians about their goals for end-of-life care.  led to an 18% increase in reported new serious illness conversations 1 month after ED GOAL, and 33% of participants had newly documented serious illness conversations on their electronic medical records within 6 months of participating. |  |  | By allowing an opportunity for patients to express values, goals, and priorities, we aim to prepare these patients at high risk of near-term mortality to be as ready as they could be if/when emergent decision-making becomes necessary. | The electronic medical record documentation of health care proxy form (62–70%) and medical order for life-sus- taining treatment form (1–11%) increased after the inter- vention within six months (interrater reliability 95%, Table 3). |
| **(Pasricha et al., 2020)** Use of the serious illness conversation guide to improve communication with surrogates of critically ill patients. A pilot study | owing to lack of funding, we did not use actors in simulation training. Rather, trainers and/or participants served as standardized patients using Ariadne Labs scripts modified for conversations with  surrogates. |  |  | Challenges to protocol adherence included 1) the requirement of family presence to complete the conversation, 2) lack of a standard process to identify eligible patients in whom an SIC had not been completed, 3) delayed and inconsistent adoption across providers, 4) apparent reluctance to administer the SIC to those less likely to die, and 5) time required to complete the conversation. | Providers reported that the program relieved their anxiety to have these types of discussions, enhanced their professional satisfaction, and gave them the perception that the conversation did not negatively impact the emotional state of the family member. |  |  |  |  | Providers reported that  the tool was easy to use, facilitated gathering of information that enhanced clinical care, helped with understanding the patient’s values and goals of care, and helped to build trust between the provider and family  Gave opportunity to align prognostic expectations early | the conversation significantly increased Surrogates understanding of their loved one’s health condition, increased their sense of control, and strengthened their relationship with the clinical team.  Overall, surrogates reported that the conversation was mostly or extremely worthwhile in 95% of cases (even when patients died during hospitalisation) | the structured communication tool elicited insights into the patients’ goals, fears and worries, sources of strength, critical abilities, and how much the patient would be willing to go through for the possibility of gaining more time | The conversation was then documented using a standardized template available within the electronic health record (EHR). |
| **(Smith, 2017)**  Advance care planning communication for young adults: a role for simulated learning | The simulation was useful and valuable to the nurses sampled. It increased their awareness and allowed them to practice ACP communication in a safe, nonthreatening environment. |  | participants expressed that ACP instruction is lacking in nursing programs. The literature on nursing and NP education in the area of palliative care and ACP communication supports that educational offerings are limited |  | After a 45- to 50-minute simulation exercise, these nurses expressed significant increases in self-confidence and skills not only to discuss ACP but also to initiate the conversation. |  |  |  |  |  |  |  |  |
| **(Wolfe et al., 2016)**  Bad news deserves better communication: A customizable curriculum for teaching learners to share life-altering Information in pediatrics | The cases are designed to illustrate themes identified in the workshop needs assessment, such as working with adolescent patients, forming therapeutic alliances under difficult circumstances, and exhibiting compassion in the face of multiple different emotional responses.  Another important lesson is that learners often find role-playing to be challenging; however, learners consistently report in follow-up feedback sessions that they find these sessions to be very helpful and appreciate practicing communication skills prior to using these skills in real-life situations. |  |  | Participants also identified several barriers and areas for improvement. Providers need more time to spend with families during these conversations. Providers need more practice and experience. During the workshop, facilitators should consider limiting how long each trainee spends with a respondent. More time talking to the parent panel during the workshop would be valuable. |  |  |  |  |  | Needs assessment revealed the following needs:  - Allowing families to maintain hope in the face of life-altering information.  - Sharing information compassionately.  - Maintaining a therapeutic alliance when medical news is bad.  - Working with adolescent and young adult patients and their parents.  - Learning how to teach these skills effectively.  longitudinal curriculum on sharing life-altering information is most effective in improving pediatrician–family communication |  |  |  |

Aaronson, E. L., Kennedy, M., Gillis-Crouch, G., Zheng, H., Jacobsen, J., Ouchi, K., Jackson, V., Ritchie, C. S., Gioiella, M. E., & Greenwald, J. L. (2023). Assessing the acceptability and feasibility of leveraging emergency department social workers' advanced communication skills to assess elderly patients' goals and values. *Journal of Palliative Medicine*, *26*(4), 517-526. <https://doi.org/10.1089/jpm.2022.0136>

Annadurai, V., Smith, C. B., Bickell, N., Berns, S. H., Kelley, A. S., Lindenberger, E., Morrison, L. J., Yuen, J. K., Egorova, N., Franco, R., Back, A. L., & Gelfman, L. P. (2021). Impact of a novel goals-of-care communication skills coaching intervention for practicing oncologists. *Journal of Palliative Medicine*, *24*(6), 838-845. <https://doi.org/10.1089/jpm.2020.0207>

Banerjee, S. C., Manna, R., Coyle, N., Penn, S., Gallegos, T. E., Zaider, T., Krueger, C. A., Bialer, P. A., Bylund, C. L., & Parker, P. A. (2017). The implementation and evaluation of a communication skills training program for oncology nurses. *Translational behavioral medicine*, *7*(3), 615-623. <https://doi.org/https://doi.org/10.1007/s13142-017-0473-5>

Berns, S. H., Camargo, M., Meier, D. E., & Yuen, J. K. (2017). Goals of care ambulatory resident education: Training residents in advance care planning conversations in the outpatient setting. *Journal of Palliative Medicine*, *20*(12), 1345-1351. <https://doi.org/10.1089/jpm.2016.0273>

Casey, M. F., Price, L., Markwalter, D., Bohrmann, T., Tsujimoto, T. M., Lavin, K., Hanson, L. C., Lin, F.-C., & Platts-Mills, T. F. (2022). Advance care planning for emergency department patients with COVID-19 infection: An assessment of a physician training program. *The American Journal of Hospice & Palliative Care*, *39*(11), 1358-1363. <https://doi.org/https://doi.org/10.1177/10499091211072850>

Chatterjee, S., Roberts, B., Ahluwalia, A. K., Wright, S., & Wu, D. S. (2022). Integrating narrative goals of care in the medical intensive care unit: Impact on educational and clinical outcomes. *American Thoracic Society Scholar*, *3*(3), 449-459. <https://doi.org/doi:https://doi.org/10.34197/ats-scholar.2022-0003IN>

Day, L. B., Saunders, S., Steinberg, L., Ginsburg, S., & Soong, C. (2022). "Get the DNR": Residents' perceptions of goals of care conversations before and after an e-learning module. *Canadian Medical Education Journal*, *13*(1), 17-28. <https://doi.org/10.36834/cmej.71956>

Doherty, M., Gujral, P., Frenette, M., Lusney, N., & van Breemen, C. (2023). The Pediatric Serious Illness Conversation Program: Understanding challenges and experiences for clinicians after advance care planning training. *Palliative & supportive care*, 1-8. <https://doi.org/doi:https://dx.doi.org/10.1017/S1478951523000500>

Epstein, R. M., Duberstein, P. R., Fenton, J. J., Fiscella, K., Hoerger, M., Tancredi, D. J., Xing, G., Gramling, R., Mohile, S., Franks, P., Kaesberg, P., Plumb, S., Cipri, C. S., Street, R. L., Shields, C., G., Back, A. L., Butow, P., Walczak, A., . . . Kravitz, R. L. (2017). Effect of a patient-centered communication intervention on oncologist-patient communication, quality of life, and health care utilization in advanced cancer: The VOICE randomized clinical trial. *Journal of the American Medical Association Oncology*, *1*(5), 92-100. <https://doi.org/doi:https://doi.org/10.1001/jamaoncol.2016.4373>

Fettig, L., Tang, Q., Newton, E., Rosario, R., Matthias, M. S., & Torke, A. M. (2022). A communication skills training workshop to improve ICU Team relational coordination about goals of care: A pilot study. *American Journal of Hospice and Palliative Care*, *39*(10), 1157-1164. <https://doi.org/10.1177/10499091211069994>

Geerse, O. P., Lamas, D. J., Sanders, J. J., Paladino, J., Kavanagh, J., Henrich, N. J., Berendsen, A. J., Hiltermann, T. J. N., Fromme, E. K., Bernacki, R. E., & Block, S. D. (2019). A qualitative study of serious illness conversations in patients with advanced cancer. *Journal of Palliative Medicine*, *22*(7), 773-781. <https://doi.org/10.1089/jpm.2018.0487>

Haley, E. M., Meisel, D., Gitelman, Y., Dingfield, L., Casarett, D. J., & O'Connor, N. R. (2017). Electronic goals of care alerts: An innovative strategy to promote primary palliative care. *Journal of Pain and Symptom Management*, *53*(5), 932-937. <https://doi.org/10.1016/j.jpainsymman.2016.12.329>

Kruser, J. M., Taylor, L. J., Campbell, T. C., Zelenski, A., Johnson, S. K., Nabozny, M. J., Steffens, N. M., Tucholka, J. L., Kwekkeboom, K. L., & Schwarze, M. L. (2017). "Best Case/Worst Case": Training surgeons to use a novel communication tool for high-risk acute surgical problems. *Journal of Pain Symptom Management*, *53*(4), 711-719 e715. <https://doi.org/10.1016/j.jpainsymman.2016.11.014>

Lagrotteria, A., Swinton, M., Simon, J., King, S., Boryski, G., Ma, I. W. Y., Dunne, F., Singh, J., Bernacki, R. E., & You, J. J. (2021). Clinicians' perspectives after implementation of the serious illness care program: A qualitative study. *Journal of the American Medical Association Network Open*, *4*(8), e2121517. <https://doi.org/10.1001/jamanetworkopen.2021.21517>

Moody, K. M., Hendricks-Ferguson, V. L., Baker, R., Perkins, S., & Haase, J. E. (2020). A pilot study of the effects of COMPLETE: A communication plan early through end of life, on end-of-life outcomes in children with cancer. *Journal of Pain Symptom Management*, *60*(2), 417-421. <https://doi.org/10.1016/j.jpainsymman.2020.03.033>

Ouchi, K., Lee, R. S., Block, S. D., Aaronson, E. L., Hasdi, a, M. A., Wang, W., Rossmassler, S., Palan Lopez, R., Berry, D., Sudore, R., Schonberg, M. A., & Tulsky, J. A. (2023). An emergency department nurse led intervention to facilitate serious illness conversations among seriously ill older adults: A feasibility study. *Palliative Medicine*, *37*(5), 730-739. <https://doi.org/doi:https://doi.org/10.1177/02692163221136641>

Pasricha, V., Gorman, D., Laothamatas, K., Bhardwaj, A., Ganta, N., & Mikkelsen, M. E. (2020). Use of the serious illness conversation guide to improve communication with surrogates of critically ill patients. A pilot study. *American Thoracic Society Scholar*, *1*(2), 119-133. <https://doi.org/10.34197/ats-scholar.2019-0006OC>

Smith, S. L. (2017). Advance care planning communication for young adults: A role for simulated learning. *Journal of Hospice and Palliative Nursing*, *19*(5), 460-467. <https://doi.org/10.1097/NJH.0000000000000373>

Wolfe, A. D., Denniston, S. F., Baker, J., Catrine, K., & Hoover-Regan, M. (2016). Bad news deserves better communication: A customizable curriculum for teaching learners to share life-altering Information in pediatrics. *MedEdPORTAL*, *12*, 10438. <https://doi.org/https://doi.org/10.15766/mep_2374-8265.10438>
